# Inhibition of PRL2 Upregulates PTEN and Attenuates Tumor Growth in *Tp53*-deficient Sarcoma and Lymphoma Mouse Models

**DOI:** 10.1158/2767-9764.CRC-23-0308

**Published:** 2024-01-02

**Authors:** Frederick Nguele Meke, Yunpeng Bai, Diego Ruiz-Avila, Colin Carlock, Jinan Ayub, Jinmin Miao, Yanyang Hu, Qinglin Li, Zhong-Yin Zhang

**Affiliations:** 1 Department of Medicinal Chemistry and Molecular Pharmacology, Purdue University, West Lafayette, Indiana.; 2 Department of Chemistry, Purdue University, West Lafayette, Indiana.; 3 Institute for Cancer Research, Purdue University, West Lafayette, Indiana.; 4 Institute for Drug Discovery, Purdue University, West Lafayette, Indiana.

## Abstract

The phosphatases of regenerating liver (PRL) are oncogenic when overexpressed. We previously found that PRL2 deletion increases PTEN, decreases Akt activity, and suppresses tumor development in a partial *Pten*-deficient mouse model. The current study aims to further establish the mechanism of PTEN regulation by PRL2 and expand the therapeutic potential for PTEN augmentation mediated by PRL2 inhibition in cancers initiated without PTEN alteration. The *TP53* gene is the most mutated tumor suppressor in human cancers, and heterozygous or complete deletion of *Tp53* in mice leads to the development of sarcomas and thymic lymphomas, respectively. There remains a lack of adequate therapies for the treatment of cancers driven by *Tp53* deficiency or mutations. We show that *Prl2* deletion leads to PTEN elevation and attenuation of Akt signaling in sarcomas and lymphomas developed in *Tp53* deficiency mouse models. This results in increased survival and reduced tumor incidence because of impaired tumor cell proliferation. In addition, inhibition of PRL2 with a small-molecule inhibitor phenocopies the effect of genetic deletion of *Prl2* and reduces *Tp53* deficiency–induced tumor growth. Taken together, the results further establish PRL2 as a negative regulator of PTEN and highlight the potential of PRL2 inhibition for PTEN augmentation therapy in cancers with wild-type PTEN expression.

**Significance:**

*Prl2* deletion attenuates *Tp53* deficiency–induced tumor growth by increasing PTEN and reducing Akt activity. Targeting *Tp53*-null lymphoma with PRL inhibitors lead to reduced tumor burden, providing a therapeutic approach via PTEN augmentation.

## Introduction

Overexpression of the phosphatase of regenerating liver (PRL) phosphatases—PRL1, PRL2, and/or PRL3, occurs in many human cancers and often correlate with metastasis or poor disease prognosis (1–4). Reports suggest that PRL overexpression could potentiate major signaling pathways that are frequently hyperactivated in cancer, such as the PI3K/Akt signaling cascade (1). This highlights the therapeutic potential for PRL inhibition in cancer. Although the cancer-promoting role of PRLs is almost certain, the mechanisms by which they promote tumorigenesis are under active investigation. To study the mechanistic role of PRL2—the most ubiquitously expressed PRL—in cancer and other physiologic processes, mice without *Prl2* gene expression were generated. These *Prl2*-null mice display impaired spermatogenesis, placental insufficiency, and abnormal hematopoietic stem cell self-renewal (5–8). These deficiencies are the result of impaired Akt signaling in the affected tissues caused by increased PTEN protein level in *Prl2*-null mice. We found that PRL2 dephosphorylates PTEN on Y336, and this dephosphorylation allows binding of the E3-ubiquitin ligase NEDD4 to PTEN for PTEN polyubiquitination and subsequent proteasomal degradation (9). PTEN is a negative regulator of the PI3K/Akt signaling pathway, in which it dephosphorylates phosphatidylinositol (3–5) trisphosphate (PIP3) to phosphatidyl inositol (4, 5) bisphosphate (PIP2) to hinder Akt activation. Thus, *Prl2* deletion promotes PTEN stabilization and accumulation, which would lead to attenuated Akt activation. PTEN is a haploinsufficiency tumor suppressor gene, and even subtle decrease in PTEN protein expression increases cancer susceptibility (10–12). In several cancers, PTEN downregulation occur in later stages or during disease recurrence. The mechanism we advanced where PRL2 downregulates PTEN protein is therefore a plausible mechanism by which PRL2 promotes tumorigenesis (9). Accordingly, we showed that *Prl2* deletion in the *Pten* heterozygous mouse model prevents spontaneous tumorigenesis through PTEN upregulation and attenuated Akt signaling pathway (9). This highlighted the potential of PRL2 inhibition as a therapeutic approach for the treatment of PTEN deficiency–induced cancers. However, based on cBioPortal cancer genomic database, more than 90% of all cancers develop with wild-type (WT) PTEN expression, which emphasizes the need to develop treatments for such cancers. Whether loss of PRL2 can still prevent tumor growth in cancers that develop independently of PTEN downregulation through PTEN augmentation is still unknown.

TP53 is an important tumor suppressor that regulates important cellular processes such as DNA repair, cell cycle, apoptosis, and metabolism (13–15). Indeed, *TP53* is the most mutated or downregulated gene in human cancers. Certain cancers, such as osteosarcomas and soft-tissue sarcomas, have a high rate of *TP53* mutation or downregulation, and novel approaches to treat cancers with *TP53* abnormalities would play a critical role to improve patient outcome (16, 17). Proposed approaches for the treatment of cancers with P53 mutations involve the use of small molecules for the restoration of WT P53 function, P53 stabilization by inhibiting its interaction with negative regulators (such as MDM2 and MDMX), and activation of P53 family members such as P73 (18–20). Although these approaches show promise in laboratory research, clinical trials exploiting these strategies show adverse effect due to off-target effect, that hinder the progress of such therapies (18). Thus, developing novel approaches for the treatment of cancers with P53 downregulation or mutations remains a high priority. Consistent with human patient studies, partial or complete deletion of *Tp53* in mice leads to the spontaneous tumorigenesis, with sarcomas and thymic lymphomas being the major cancers observed, respectively (21–23). Thus, providing adequate models to evaluate novel approaches for the treatment of cancers driven by P53 downregulation is highly needed. We know that *Prl2* deletion in WT mice can elevate PTEN (5, 7, 24), but it remains unclear whether this can inhibit cancers that develop independently of PTEN downregulation. We hypothesized that PTEN augmentation induced by PRL2 inhibition in cancers with reduced P53 expression could serve as a promising therapeutic approach. Indeed, we found patients with sarcoma with TP53 mutations have higher *PRL2* mRNA expression and lower PTEN protein level, supporting our hypothesis that targeting PRL2 to enhance PTEN level could serve as a therapeutic approach for cancer treatment in *TP53*-driven cancer. By using the well-established P53 deficiency mouse models (heterozygous and homozygous *Tp53* deletion), we sought to not only further establish the mechanism of PTEN regulation by PRL2 but also assess the therapeutic potential of PRL2 inhibition for PTEN augmentation therapy in the treatment of cancers that develop without PTEN alteration.

Through bioinformatics analysis, we show that *PRL2* mRNA expression negatively correlates with survival in patients with sarcoma, and positively correlates with cell proliferation markers in these patients. In addition, we found that only a small portion of patients with sarcoma have PTEN mutation, suggesting such cancers are suitable for PTEN augmentation therapy. We show that in both *Tp53* heterozygous and *Tp53*-null mouse models, deletion of *Prl2* significantly improves survival and delays tumor growth. Mechanistically, we confirmed increased PTEN protein level and reduced Akt activation in sarcomas and lymphomas obtained from *Prl2*-null mice. The reduced growth was determined to be a result of reduced cell proliferation, but interestingly, we observed no change in apoptosis in both models. In addition, we show that pharmacologic inhibition of PRL2 with a small-molecule PRL inhibitor—Cmpd-43 (24), significantly hinders tumor growth in *Tp53*-null mice. Our findings: (i) further substantiate the mechanism of PTEN downregulation by PRL2 *in vivo*, (ii) demonstrate the potential of PRL2 inhibition for PTEN augmentation therapy in cancers that develop with WT PTEN expression and P53 mutations, and (iii) establish the efficacy of small-molecule inhibition of PRLs in an *in vivo* mouse tumor model for the first time. Together, the results suggest that pharmacologic inhibition of PRL2 could serve as a novel therapeutic strategy to increase PTEN protein levels, thereby obliterating P53 deficiency–induced malignancies.

## Materials and Methods

### Reagents and Antibodies

Tamoxifen (CAS 10540-29-1) was obtained from as a powder from Cayman Chemicals (catalog no. 13258). The following antibodies used for FACS analysis were obtained from BioLegend: CD4-488 (BioLegend, catalog no. 100423, RRID: AB_389302), CD8a-PE (BioLegend, catalog no. 100708, RRID: AB_312747), CD45-PeCy7 (BioLegend, catalog no. 103114, RRID: AB_312979), CD25-BV605 (BioLegend, catalog no. 102035, RRID: AB_11126977), CD44-647 (BioLegend, catalog no. 103039, RRID: AB_10895752). Primary antibodies used for Western blotting were: PTEN (Cell Signaling Technology, catalog no. 9188, RRID: AB_2253290), AKT (Cell Signaling Technology, catalog no. 9272, RRID: AB_329827), p-AKT pS473 (Cell Signaling Technology, catalog no. 4060, RRID:AB_2315049), mTOR (Cell Signaling Technology, catalog no. 2972, RRID: AB_330978), p-mTOR pS2448 (Cell Signaling Technology, catalog no. 2971, RRID:AB_330970), β-actin (Santa Cruz Biotechnology, catalog no. sc-47778, RRID: AB_626632), PARP (Cell Signaling Technology, catalog no. 9542, RRID: AB_2160739), Caspase 3 (Cell Signaling Technology, catalog no. 14220, RRID: AB_2798429), p-BAD pS136 (Cell Signaling Technology, catalog no. 4366, RRID: AB_10547878), PCNA (Cell Signaling Technology, catalog no. 2586, RRID:AB_2160343), FoxO1 (Cell Signaling Technology, catalog no. 2880, RRID:AB_2106495), p-FoxO1/3a/4 pT24/32/28 (Cell Signaling Technology, catalog no. 2599, RRID:AB_2106814), GSK3-alpha/beta (Cell Signaling Technology, catalog no. 5676, RRID:AB_10547140), p-GSK3alpha/beta pS21/9 (Cell Signaling Technology, catalog no. 9331, RRID:AB_329830), ERK1/2 (Cell Signaling Technology, catalog no. 4696, RRID:AB_390780), p-ERK1/2 pT202/pY204 (Cell Signaling Technology, catalog no. 4370, RRID:AB_2315112), BAX (Cell Signaling Technology, catalog no. 2772, RRID:AB_10695870), p27 (Santa Cruz Biotechnology, catalog no. sc-528, RRID:AB_632129), p21 (Santa Cruz Biotechnology, catalog no. sc-6246, RRID:AB_628073), cyclin D1 (Santa Cruz Biotechnology, catalog no. sc-20044, RRID: AB_627346), 4E-BP1 (Santa Cruz Biotechnology, catalog no. sc-9977, RRID: AB_626621), p-4E-BP1/2/3 (Santa Cruz Biotechnology, catalog no. sc-271947, RRID:AB_10709575), PUMA (Thermo Fisher Scientific, catalog no. PA5-20007, RRID: AB_11153580), p53 (Leica Biosystems, catalog no. NCL-L-p53-CM5p, RRID: AB_2895247) Secondary antibodies used were: Anti-rabbit IgG, horseradish peroxidase (HRP)-linked (Cell Signaling Technology, catalog no. 7074, RRID: AB_2099233), and Anti-mouse IgG, HRP-linked (Cell Signaling Technology, catalog no. 7076, RRID: AB_330924).

### Transgenic Mice

This study utilizes commercially available C57BL/6J Rosa26-CreER^T2^ (Strain# 008463, RRID: IMSR_JAX:008463) and C57BL/6J *Tp53^flox^* (Strain# 008462, RRID: IMSR_JAX:008462) mice from Jackson Labs. A *Prl2^flox^* mouse was generated within C57BL/6J strain mice in-house using CRISPR/Cas9 recombination as recently described by Carlock and colleagues (25). Comparable numbers of male and female mice were enrolled in each study. Animals were housed and cared for in accordance with Purdue University Institutional Animal Care and Use Committee guidelines and all experiments conducted followed our approved protocol (protocol number 1511001324).

### Genotyping

DNA was isolated from 1 mm tail snips taken from mouse pups during weaning. Tails snips were boiled at 95°C for 20 minutes in a solution of 25 mmol/L NaOH and 200 µmol/L ethylenediaminetetraacetic acid (EDTA) buffered to pH 12. The solution was cooled to room temperature, and then neutralized by the addition of equal volume 40 mmol/L Tris-HCl, pH 5. The following primer sets were used to identify transgenes: Rosa26 WT forward: CTG GCT TCT GAG GAC CG, reverse: CCG AAA ATC TGT GGG AAG TC; Rosa26-CreER^T2^ forward: CGT GAT CTG CAA CTC CAG TC, reverse: AGG CAA ATT TTG GTG TAC GG; *Tp53^flox^* forward: GGT TAA ACC CAG CTT GAC CA, reverse: GGA GGC AGA GAC AGT TGG AG; *Prl2^flox^* forward: CAC ACA CTT AAG TAA GTA CCT GGT TGG, reverse: CCA ATC ATC TAC TAT CTG ATT AGG G. PCR reaction mixtures were made using GoTaq Flexi DNA Polymerase (Promega, catalog no. M8295), and cycling protocols were performed according to Jackson Labs recommendation. The in-house *Prl2^flox^* cycling conditions were as follows: 95°C-5 minutes, (95°C-30 seconds, 57°C-45 seconds, 72°C-1 minute) × 34, 72°C-5 minute, 4°C-hold.

### Tamoxifen Treatment

Tamoxifen powder was dissolved in corn oil (Sigma-Aldrich, catalog no. C8267) at a stock concentration of 20 mg/mL by rocking overnight in the dark at 37°C. This stock solution was then filtered through a 70 µm mesh filter (Falcon, catalog no. 352350) and stored at 4°C until necessary. Animals were given intraperitoneal injections of tamoxifen at the concentration indicated for each protocol using sterile 25G needles (BD, catalog no. 305125).

### Cmpd-43 Treatment

A 3 mg/mL solution of Cmpd-43 (24) in PBS with 10% DMSO was obtained. On week 9 after tamoxifen treatment, a volume (of the 3 mg/mL Cmpd-43 solution) equal to 10 times the mouse's mass was injected intraperitoneally daily to achieve the 30 mg/kg daily dosage of Cmpd-43. This treatment was done daily for 3 weeks.

### Sample Preparation for Western Blot Analyses

Tissue samples were homogenized on dry ice using a lysis buffer made of 50 mmol/L Tris-HCl, 150 mmol/L NaCl, 10% glycerol, and 0.5% Triton X-100 containing a cocktail of protease inhibitors (Roche, catalog no. 04693132001) and phosphatase inhibitor cocktail (bimake.com, catalog no. B15001). Protein concentration was quantified by Bradford assay using the Coomassie Plus Protein Assay kit (Thermo Fisher Scientific, catalog no. 23236). Samples were electrophoresed on 7.5%–15% SDS-PAGE gels using the Bio-Rad Mini-PROTEAN gel casting system. Samples were transferred to nitrocellulose membranes, blocked with 5% w/v BSA in Tris-buffered saline with Tween 20 (TBST) and then incubated with primary antibodies at a 1:1,000–1:2,000 dilution in TBST overnight at 4°C. Membranes were washed and then incubated with HRP-conjugated secondary antibodies at a 1:3,000–1:5,000 dilution in TBST overnight at 4°C. Membranes were washed and then visualized using SuperSignal West Pico (Thermo Fisher Scientific, catalog no. 34580), or SuperSignal West Femto (Thermo Fisher Scientific, catalog no. 34095) Chemiluminescent Substrate as necessary.

### Tissue Sectioning, Staining, and Microscopy

Fixed tumors were stored in 75% ethanol then submitted to the Purdue University histology laboratory where they were embedded in paraffin, sectioned, and stained for proliferating cell nuclear antigen (PCNA; Abcam, catalog no. ab29, RRID: AB_303394) and cleaved caspase 3 (Cell Signaling Technology, catalog no. 9661S) using standard protocol.

### Sample Preparation for FACS Analysis

To perform FACS analysis, thymic lymphoma samples were first reduced to single-cell suspensions by compressing the tissue through a 70 µm filter (Falcon, catalog no. 352350) into cold 1x PBS supplemented with 5 mmol/L EDTA using the plunger of a syringe (HSW, catalog no. 4010-200V0). Sample were strained through a second filter and then resuspended in cold blocking buffer made of 5% BSA, 0.5 mmol/L EDTA, and a 1:1,000 dilution of CD16/32. Whole blood collected via cardiac puncture was prepared by lysing red blood cells in a buffer made with 155 mmol/L NH_4_Cl, 12 mmol/L NaHCO_3_, and 0.1 mmol/L EDTA for 1 minute, washing in 1x PBS, and then blocking buffer. Samples were then incubated with fluorophore conjugated antibodies. Samples were incubated with antibodies at 1:600 dilution in blocking buffer on ice for 2 hours. The following antibody combinations were used for population analysis: CD45, CD4, CD8, CD44, CD25. The gating strategy is shown in Supplementary Fig. S1. After staining samples were washed and resuspended in 5% BSA, 0.5 mmol/L EDTA solution, then added to 5 mL polystyrene tube for analysis (Falcon, catalog no. 352235). Sample data were acquired using a BD LSRFortessa Cell Analyzer, and results were analyzed using FlowJo (RRID: SCR_008520).

### The Cancer Genome Atlas Analysis

The Cancer Genome Atlas (TCGA; RRID: SCR_003193) level 3 RNAseqV2 gene expression, reverse phase protein array (RPPA), and clinical data of primary patient clinical samples from Sarcoma (SARC, *n* = 261) were downloaded from the Broad Institute's Firehose (https://gdac.broadinstitute.org/). Patients were separated into two subgroups (Tp53 WT and TP53 mutated) based on Tp53 mutation status or (low PRL2 and high PRL2) based on their PRL2 mRNA expression levels using a cutoff on the z scores (|z| ≥ 1) for PRL2 mRNA, PTEN, and p53 RPPA analysis and Kaplan–Meier survival analysis. The correlation coefficients between PRL2 mRNA and cell cycle–related genes (Cyclin E1, FoxM1, PCNA, Cyclin B1) were measured by Pearson correlation coefficients analysis. The number of each analysis was also affected by the data availability.

### Data Availability

The data generated in this study are available upon request from the corresponding author.

### Statistical Analysis

Western blot data were quantified for statistical analysis using ImageJ software (RRID: SCR_003070). Protein abundance was determined as black pixel intensity after subtracting background. Protein concentration was determined relative to loading control concentration. Phosphorylated protein concentration was determined by concentration relative to total protein. Protein concentration datasets were analyzed by the Student *t* test. Tumor weights were also compared using the Student *t* test. Survival analysis was performed using the Kaplan–Meier log-rank test. Error bars in figures indicate SEM. *P* values less than 0.05 were not considered statistically significant. Analysis was performed on all datasets using Microsoft Excel (RRID: SCR_016137) to identify outliers which were then excluded from all figures presented in this article. Outliers were defined as datapoints below the lower limit = 1.5*IQR − Q1 or above the upper limit = 1.5*IQR + Q3. IQR = interquartile range, Q1 = 1st quartile, Q3 = 3rd quartile.

### Study Approval

All procedures performed on mice were approved by the Purdue Institutional Animal Care and Use Committee.

## Results

### PRL2 Expression Negatively Correlates with Survival and PTEN Protein Expression in Human Patients with Sarcoma

**FIGURE 1:**
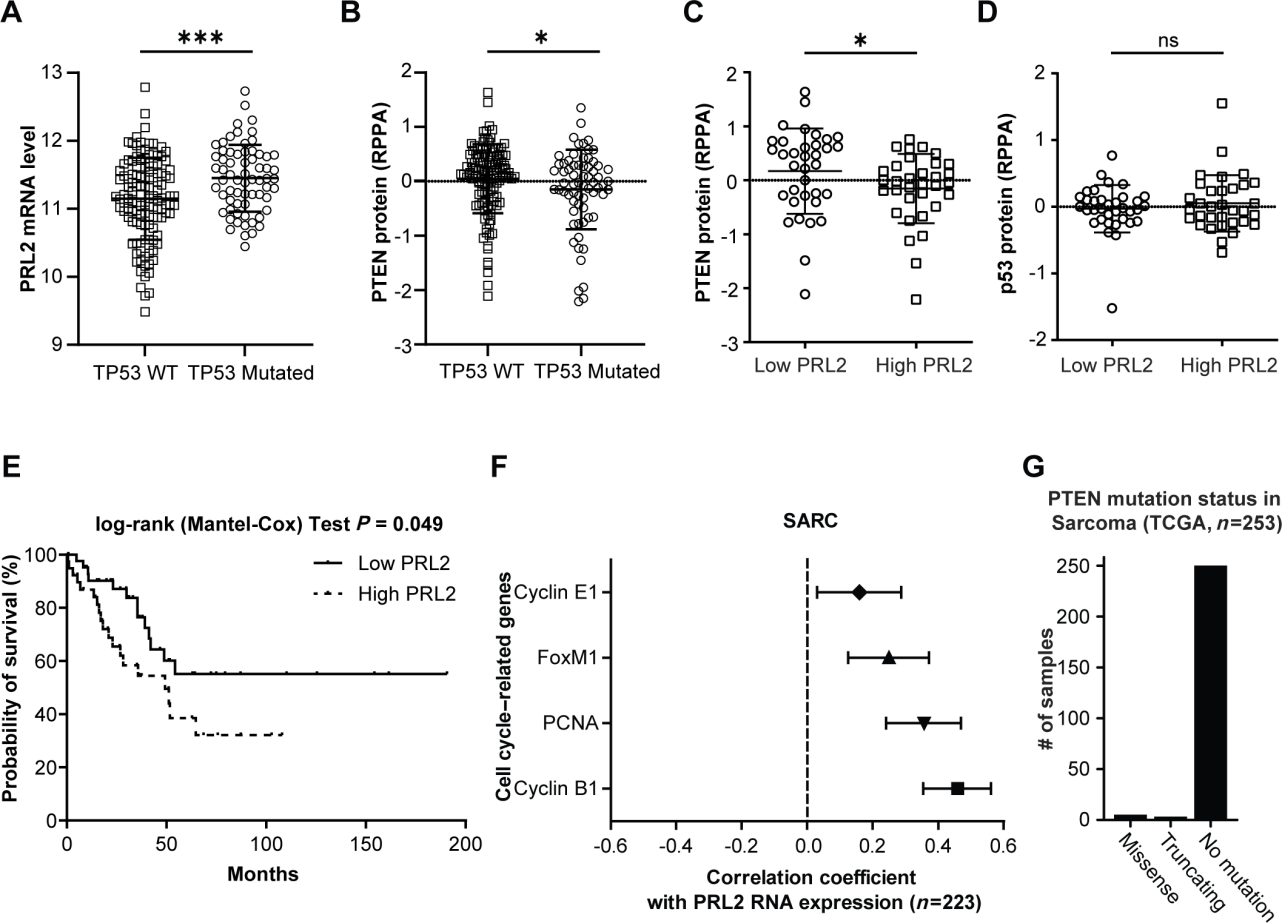
PRL2 expression inversely correlates with PTEN and survival in human patients with sarcoma. **A,** Scatter plot of PRL2 mRNA data showing the increased expression of PRL2 mRNA in patients with TCGA SARC with TP53 mutations. Patient samples were divided into TP53 WT group (*n* = 159) and TP53 mutated group (*n* = 82) based on TP53 mutation status, and PRL2 mRNA level of the two groups were plotted. **B,** Scatter plot of RPPA data of PTEN protein showing the decreased expression of PTEN protein in patients with SARC with TP53 mutations. Patient samples were divided into TP53 WT group (*n* = 122) and TP53 mutated group (*n* = 67) based on TP53 mutation status, and PTEN protein level of the two groups were plotted. **C,** Scatter plot of RPPA data showing the reverse correlation between the mRNA levels of PRL2 and protein levels of PTEN in patients with TCGA SARC. Patient samples were divided into low PRL2 (*n* = 35) and high PRL2 (*n* = 33) groups based on the PRL2 mRNA level, and then the PTEN protein level of the two groups were plotted. **D,** Scatter plot of RPPA data showing no correlation between the mRNA levels of PRL2 and protein levels of p53 in patients with TCGA SARC. Patient samples were divided into low PRL2 (*n* = 35) and high PRL2 (*n* = 33) groups based on the PRL2 mRNA level, and then the p53 protein level of the two groups were plotted. **E,** Patients with high PRL2 mRNA level (*n* = 43) have significantly reduced probability of survival than that with low PRL2 mRNA level (*n* = 39) in SARC by Kaplan–Meier survival analysis. **F,** Correlation between PRL2 mRNA and cell cycle–related gene expression in TCGA sarcoma patient samples (*n* = 223). **G,** PTEN mutation status in TCGA sarcoma patient samples (*n* = 253). For statistics, *, *P* < 0.05. ***, *P* < 0.001.

*TP53* is the most mutated or downregulated tumor suppressor gene in human cancers, thus the development of therapeutic strategies for cancers with *TP53* mutations or downregulations remains critical for cancer treatment. In this work, we propose that PRL2 inhibition for PTEN augmentation can impede tumor growth in *Tp53* deficiency–driven cancers. *Tp53* deficiency in mouse models primarily leads to sarcomas and lymphomas (23, 26–28). We therefore decided to analyze sarcoma and lymphoma patient samples from TCGA database (https://gdac.broadinstitute.org/), but only sarcoma patient samples were available. We first examined the *PRL2* mRNA expression in patients with sarcoma with or without *TP53* mutations. We found that patients with *TP53* mutation have higher *PRL2* mRNA expression (Fig. 1A). Meanwhile, these patients also showed reduced PTEN protein level (Fig. 1B). These data support our hypothesis that targeting PRL2 to enhance PTEN level could serve as a therapeutic approach for cancer treatment in *TP53* mutant–driven cancer. We found that *PRL2* mRNA expression negatively correlates with PTEN protein levels and has no correlation with P53 protein. This is consistent with our previous findings that PRL2 promotes PTEN degradation (9). We also found that high *PRL2* expression significantly reduced survival in these patients (Fig. 1C–E). Partial reduction in PTEN expression is sufficient to increase cancer risk or severity (10, 11), thus PTEN degradation resulting from PRL2 overexpression in these cancers could explain the worse patient outcome. Moreover, we found that high *PRL2* expression in these tumors positively correlates with the expression of cell cycle–related genes that would promote tumor proliferation (Fig. 1F). To use PRL2 inhibition for PTEN augmentation therapy, the targeted cancers need to express WT PTEN. Increasing the expression of an inactive mutant of PTEN would not provide any antitumor benefits. We show that only a small portion of sarcomas have PTEN mutations (Fig. 1G), meanwhile the incidence of P53 mutations in sarcomas was reported to vary between 10% and 80% (16). Taken together, the results from this study and the bioinformatics analysis supports targeting PRL2 therapeutically for the treatment of P53-deficient sarcomas with WT PTEN expression, for PTEN augmentation cancer therapy.

### PRL2 Deletion Significantly Improves Survival in Tp53 Deficiency Mouse Models

**FIGURE 2:**
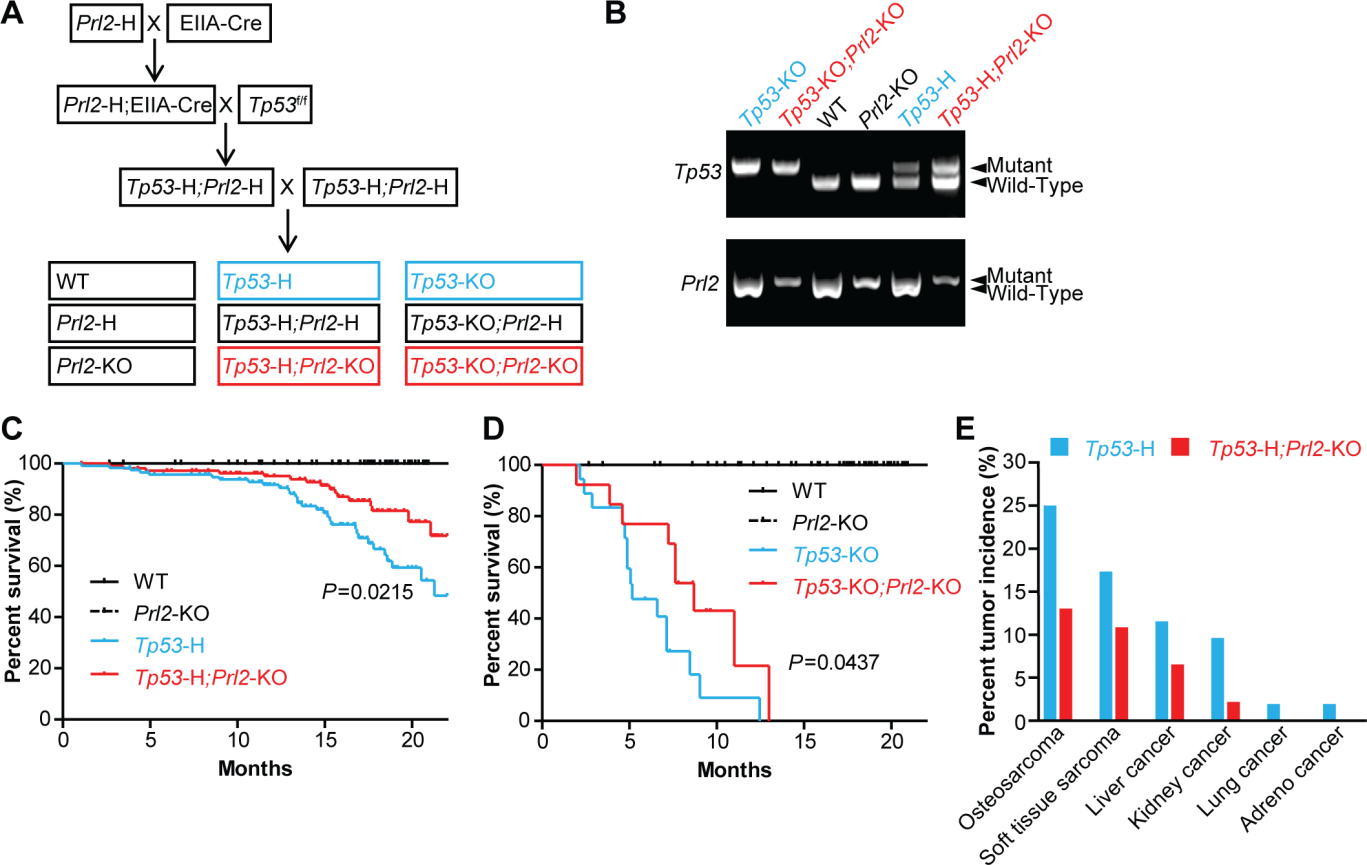
*Prl2* deletion enhances survival in constitutive *Tp53* deficiency mouse models. **A,** Mating strategy for the generation of constitutive *Tp53*-deficient mice with or without constitutive *Prl2* deletion. **B,** PCR genotyping to identify different genotypes in the study. Kaplan–Meier survival plot for overall survival in constitutive *Tp53-H* (**C**) and *Tp53-KO* (**D**) backgrounds with or without constitutive *Prl2* deletion. **E,** Bar graphs representing percent tumor incidence in *Tp53*-H background with or without *Prl2* deletion. *P* values are indicated in figures and a *P* value <0.05 is considered statistically significant. WT *n* = 44, *Prl2-KO n* = 31, *Tp53-H n* = 118, *Tp53-H; Prl2-KO n* = 108.

We previously showed that constitutive *Prl2* deletion in the context of *Pten* heterozygosity increased PTEN protein levels in both normal tissues and tumors that resulted from the PTEN deficiency (9). In this study, we wanted to investigate whether PTEN augmentation that results from PRL2 inhibition can hinder the growth of tumors that develop with WT PTEN expression. To achieve this, we decided to use *Tp53* deficiency models for spontaneous tumor development. We first generated the constitutive *Tp53* heterozygous (*Tp53-H*) and *Tp53* heterozygous; *Prl2*-knockout (*Tp53-H*; *Prl2-KO*) mice to determine the impact of PRL2 loss in the context of partial loss of P53 expression (Fig. 2A). The genotype of these mice was confirmed by PCR (Fig. 2B). As expected, *Tp53-H*; *PRL2-KO* mice had significantly improved survival compared with the *Tp53-H* control (50% survival for *Tp53-H* approximately 21.5 weeks vs. *Tp53-H*; *PRL2-KO* undetermined within timeframe of study, *P* = 0.0215; Fig. 2C). We also generated constitutive *Tp53*-knockout (*Tp53-KO*) and *Tp53*-knockout; *Prl2*-knockout (*Tp53-KO*; *Prl2-KO*) mice to assess how PRL2 affects tumorigenesis in the *Tp53-KO* background (Fig. 2A and B). Again, *Tp53-KO; Prl2-K*O mice also displayed improved survival compared the *Tp53-KO* control mice (5.2 vs. 8.7 weeks, *P* = 0.0437; Fig. 2D). Sarcomas were the most common tumors observed in *Tp53-H* background mice as expected from previous reports (23, 29), and deletion of *Prl2* significantly reduced the incidence of sarcomas in these mice (Fig. 2E). These data further support our hypothesis that deletion of *Prl2* leads to improved tumor-free survival in constitutive *Tp53* deficiency–driven tumors.

**FIGURE 3:**
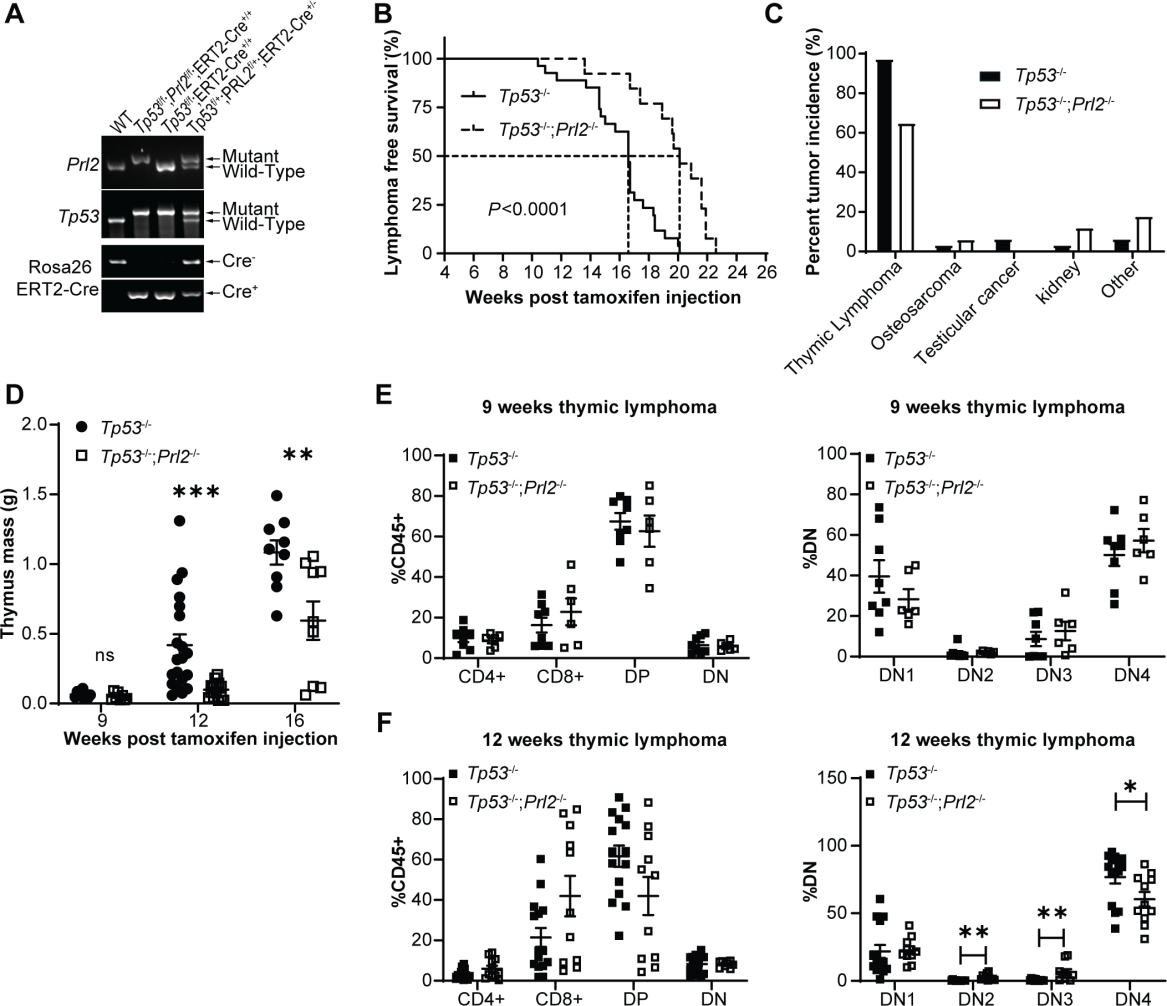
*Prl2* deletion enhances survival and hinders tumor growth rate in tamoxifen-inducible *Tp53^−^^/^^−^* mouse but does not affect tumor initiation. **A,** PCR genotyping to identify different genotypes in the study. **B,** Kaplan–Meier survival plot for thymic lymphoma-free survival in tamoxifen-inducible *Tp53^−^^/^^−^ n* = 43 and inducible *Tp53^−^^/^^−^*; *Prl2^−^^/^^−^ n* = 33. **C,** Bar graph representing percent tumor incidence in inducible *Tp53^−^^/^^−^* background with or without inducible *Prl2* deletion. Dot plots representing: The mass of thymus/thymic lymphoma over time in inducible *Tp53^−^^/^^−^ n* = 9–20 per timepoint and inducible *Tp53^−^^/^^−^*; *Prl2^−^^/^^−^ n* = 8–14 per timepoint (**D**). **E,** Thymocyte population ratios 9 weeks after inducing *Tp53* deletion with or without *Prl2* deletion. **F,** Thymocyte population ratios 12 weeks after inducing *Tp53* deletion with or without *Prl2* deletion. DP: Double positive (CD4+ CD8+). DN: Double negative (CD4− CD8−). log-rank test performed for Kaplan–Meier survival curves and Student *t* test performed for all other analysis. Data are shown as mean ± SEM. *, *P* < 0.05; **, *P* < 0.01; ***, *P* < 0.001.

Constitutive *Tp53-KO* mice show early tumor development with a median survival of approximately 4 months, with low incidence of birth defects (23, 26, 30). In addition to the constitutive *Tp53-KO* mice, we also generated tamoxifen-inducible *Tp53*-knockout (*Tp53^−^^/^^−^*) and *Tp53*-knockout; *Prl2*-knockout (*Tp53^−^^/^^−^*; *Prl2^−^^/^^−^*) mice for controlled induction of tumorigenesis. This would circumvent potential developmental defects and pre-adulthood tumorigenesis and allow us to contrast the constitutive and inducible models. Floxed-*Tp53* mice (*Tp53^flox/^^flox^*) mice were obtained commercially, and we generated our own floxed-*Prl2* (*Prl2^flox/^^flox^*) mice as described previously (25). We first bred the *Prl2^flox/^^flox^* mice with the tamoxifen-inducible ERT2-Cre^+/+^ mice to generate *Prl2^flox/^^flox^*; ERT2-Cre^+/+^ mice (Supplementary Fig. S2A). Our *Prl2^flox/^^flox^*; ERT2-Cre^+/+^ mice were then bred with control *Tp53*^flox/flox^; ERT2-Cre^+/+^ mice to generate our experimental *Prl2^flox/^^flox^*; *Tp53^flox/^^flo^*^x^; ERT2-Cre^+/+^ mice (Supplementary Fig. S2A), for which the genotyping was confirmed by PCR (Fig. 3A). To bypass major postnatal development, 8 to 10 weeks old adult mice were given tamoxifen intraperitoneally for 5 consecutive days to induce *Tp53* and/or *Prl2* whole-body deletion to generate the control *Tp53^−^^/^^−^* and experimental *Tp53^−^^/^^−^*; *Prl2^−^^/^^−^* mice, which were monitored for tumor development and survival (Supplementary Fig. S2B). Following tamoxifen treatment, we found that the experimental *Tp53^−^^/^^−^*; *Prl2^−^^/^^−^* mice had a significant increase in survival compared with the control *Tp53^−^^/^^−^* mice (20.9 vs. 16.6 weeks, *P* < 0.0001; Fig. 3B). On the other hand, the primary tumor type observed after tamoxifen treatment in *Tp53^−^^/^^−^* mice were thymic lymphomas (98% incidence), and deletion of *Prl2* in this model drastically reduced the incidence of thymic lymphomas (∼62%; Fig. 3C). Prior to tamoxifen treatment, these mice had normal PRL2 and P53 expression in the thymus, and deletion of *Prl2* and/or *Tp53* in the thymus was confirmed after tamoxifen treatment (Supplementary Fig. S2C).

Loss of P53 in mice leads to dysregulation in T-cell development and promotes the selection and expansion of lymphoblasts which results in significant increase in thymic mass (21, 29). Because we have temporal control on gene deletion, we assessed the growth rate of thymic lymphomas in our model. Our results show that simultaneous inducible deletion of *Prl2* and *Tp53* significantly hinders thymic lymphoma growth rate (*Tp53^−^^/^^−^* vs. *Tp53^−^^/^^−^*; *Prl2^−^^/^^−^* at 12 weeks 0.419 g vs. 0.098 g, *P* = 0.0017; at 16 weeks 1.083 g vs. 0.594 g, *P* = 0.0085; Fig. 3D). Interestingly, there was a modest albeit insignificant reduction in tumor size in the *Tp53^−^^/^^−^*; *Prl2^−^^/^^−^* mice at death. The tumor growth is limited to the size of the thoracic cavity, and thus both groups are expected to have comparable tumor size when death is because of the thymic lymphoma. Analysis of thymic lymphomas using flow cytometry showed thymic lymphoma onset in some mice as early as 9 weeks after tamoxifen treatment, albeit normal thymus size and morphology at this time for both groups. At 12-weeks after tamoxifen treatment, we observed depletion of the DN2 and DN3 thymocyte populations along with thymic enlargement (Fig. 3E and F), in agreement with previous models of thymic lymphomagenesis (31, 32). DN2 and DN3 thymocyte depletion was observed to a lesser extent at 12 weeks in the *Tp53^−^^/^^−^*; *Prl2^−^^/^^−^* mice, which further highlights hindered thymic lymphoma development upon *Prl2* deletion. Thymocyte population distribution in WT mice is shown in Supplementary Fig. S2D. Taken together, these results support that loss of PRL2 significantly hinders tumorigenesis in both constitutive and inducible P53 deficiency models. In addition, we determined the tumor growth rate in the inducible *Tp53^−^^/^^−^* tumor model, which will allow us to assess the therapeutic efficiency of pharmacologic PRL2 inhibition.

### Deletion of PRL2 in Tp53 Deficiency Models Inhibits Tumor Cell Proliferation but not Apoptosis

**FIGURE 4:**
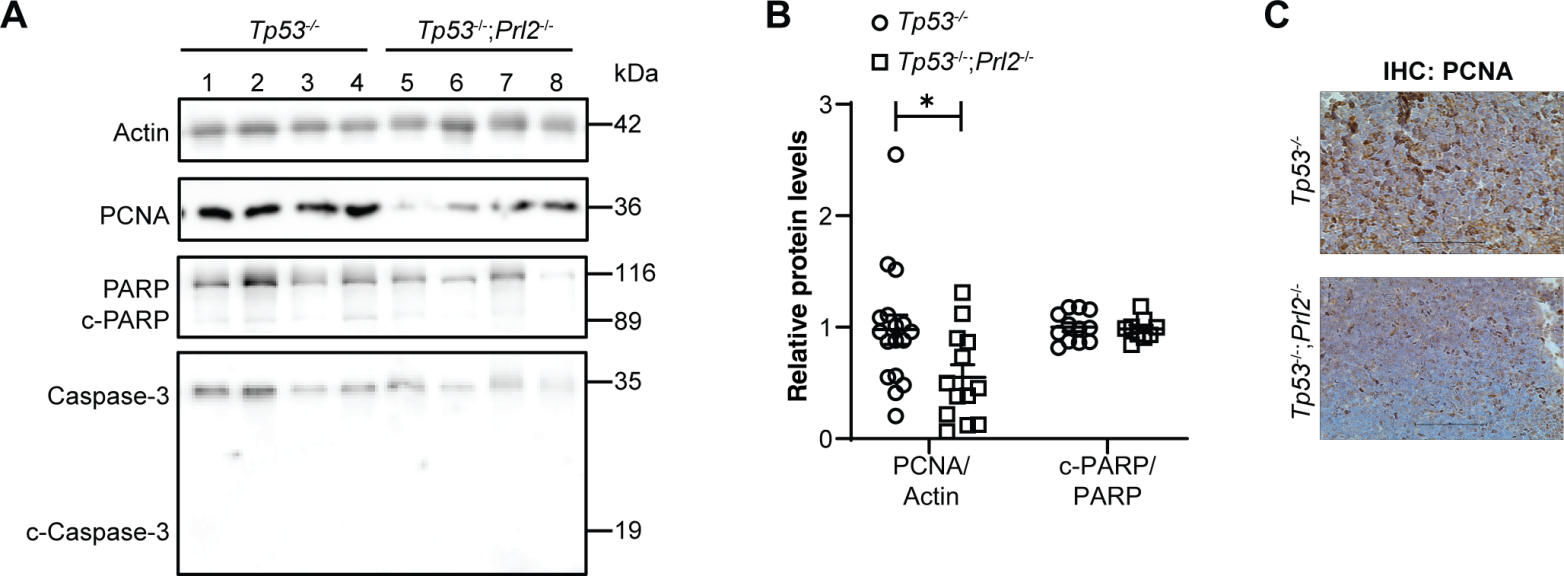
*Prl2* deletion inhibits tumor cell proliferation but not apoptosis in tamoxifen-inducible *Tp53^−^^/^^−^* mice. **A,** Representative Western blot analysis for proliferation marker PCNA and apoptosis markers c-PARP and c-caspase 3 from thymic lymphoma samples obtained 12 weeks after tamoxifen treatment. **B,** Dot plots for quantification of proliferation and apoptosis markers relative to actin and total protein control respectively for Western blots in A. Data are shown as mean ± SEM. *Tp53^−^^/^^−^ n* = 12–20, *Tp53^−^^/^^−^*; *Prl2^−^^/^^−^ n* = 11–14. **C,** Representative IHC stain using PCNA marker to compare proliferation in inducible *Tp53^−^^/^^−^* and *Tp53^−^^/^^−^*; *Prl2^−^^/^^−^* thymic lymphomas. *, *P* < 0.05.

Our results suggest that tumor growth rate is significantly reduced because of *Prl2* deletion in our *Tp53* deficiency models. To determine whether this is a result of changes in tumor cell proliferation and/or apoptosis, we assessed the expression of proliferation and apoptotic markers in the thymic lymphomas of both control and experimental mice 12 weeks after tamoxifen treatment. We used PCNA as a proliferation marker, and through both immunoblotting and IHC, we observed a significant 43% reduction in thymic lymphoma proliferation in *Tp53^−^^/^^−^*; *Prl2^−^^/^^−^* mice compared with *Tp53^−^^/^^−^* controls (Fig. 4A–C). This observation is consistent with our previous report that *Prl2* deletion reduces proliferation in the PTEN heterozygous tumor model (9). The tumor suppressor P53 is a major regulator of apoptosis, and we previously showed that deletion of *Prl2* in the PTEN heterozygous model increases tumor cell apoptosis in a P53-dependent manner (9). Because of complete loss of *Tp53* in the tamoxifen-inducible *Tp53^−^^/^^−^* model, we anticipated that *Prl2* deletion would have no effect on apoptosis. Immunoblotting for cleaved-PARP and cleaved-caspase 3 as markers for apoptosis confirmed that there is no difference in apoptosis between the *Tp53^−^^/^^−^*; *Prl2^−^^/^^−^* and *Tp53^−^^/^^−^* mice (Fig. 4A and B). Interestingly, similar results were observed when comparing sarcomas obtained from *Tp53-H; Prl2-KO* with those derived from *Tp53-H* mice, where we observed a significant 34% reduction in tumor proliferation and no change in apoptosis (Supplementary Fig. S3A–S3C). Our results suggest that loss of PRL2 in *Tp53* deficiency–derived tumors inhibits tumor growth through downregulation of tumor cell proliferation, with no effect on apoptosis.

### Loss of PRL2 in TP53-deficient Tumors Attenuates Tumor Growth through PTEN Augmentation and Inhibition of Akt Signaling

**FIGURE 5:**
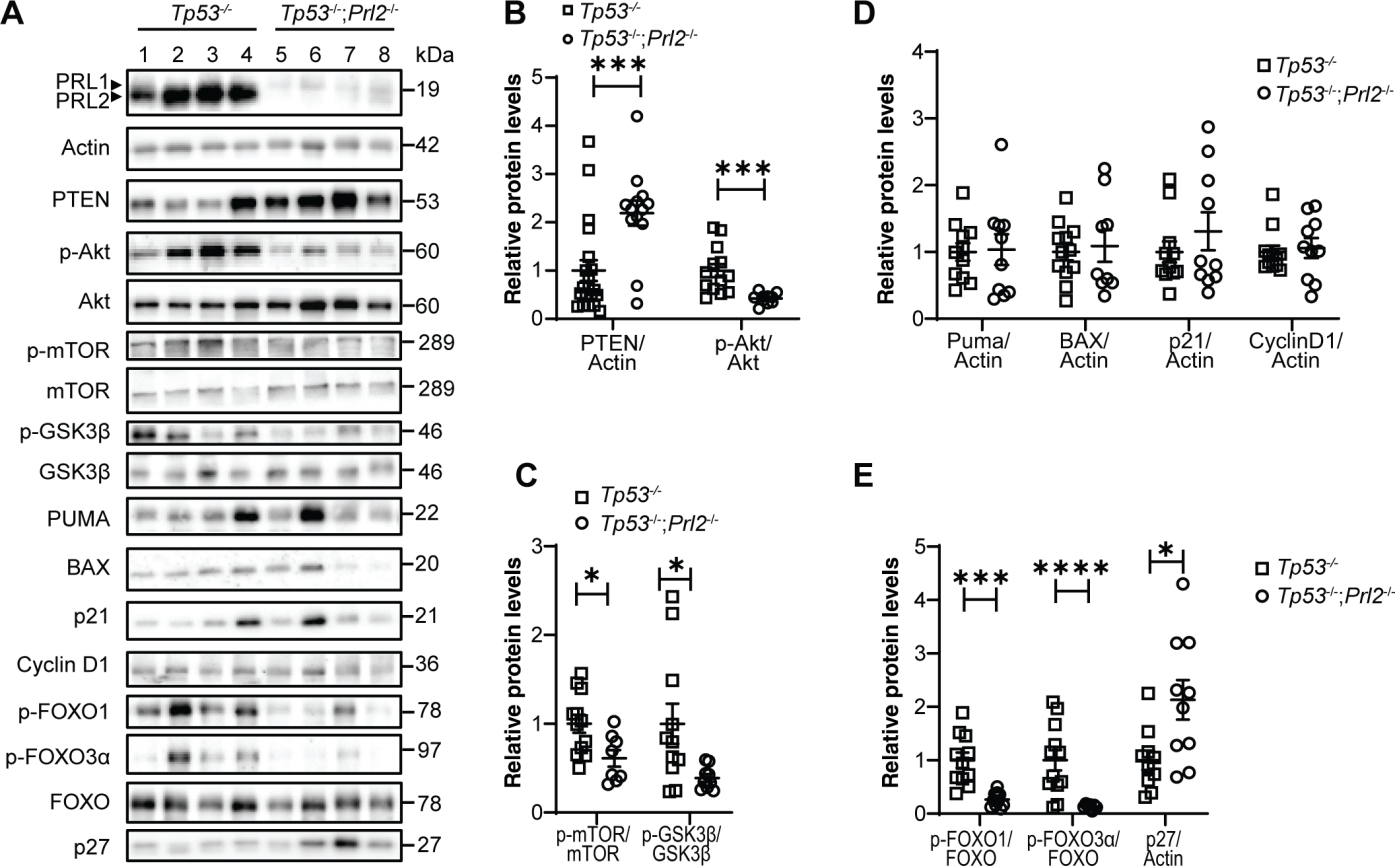
*Prl2* deletion upregulates PTEN to inhibit Akt signaling and hinder tumor cell proliferation in inducible *Tp53^−^^/^^−^* mice. **A,** Representative Western blot analysis from inducible *Tp53^−^^/^^−^* and inducible *Tp53^−^^/^^−^*; *Prl2^−^^/^^−^*–derived thymic lymphomas to determine expression of PTEN, PUMA, BAX, p21, cyclin D1, p27, as well as the expression and activation of Akt and Akt targets. The actin loading control shown is the same as that in Fig. 4A. **B,** Dot plot for quantification of PTEN and Akt activation. **C,** Dot plot for quantification of Western blot analysis for the activation of Akt targets mTOR and GSK3β. **D,** Dot plot for quantification of the expression of Tp53 targets PUMA, BAX, p21, and cyclin D1. **E,** Dot plot for quantification of FoxOs activation and p27 expression. Data shown as mean ± SEM for samples obtained 12 weeks after tamoxifen treatment, *Tp53^−^^/^^−^ n* = 10–20, *Tp53^−^^/^^−^*; *Prl2^−^^/^^−^ n* = 10–14; *, *P* < 0.05; ***, *P* < 0.001; ****, *P* < 0.0001.

Our *Tp53^−^^/^^−^*; *Prl2^−^^/^^−^* and *Tp53^−^^/^^−^* mice have WT PTEN gene, and we showed that inducible *Prl2* deletion in the thymus and other tissues also leads to higher PTEN protein level in these tissues (25). PTEN is a major negative regulator of the PI3K/Akt signaling pathway and we thus hypothesized that increased PTEN protein level in developing thymic lymphomas inhibits Akt signaling to hinder the growth of these tumors as observed. As expected, by performing immunoblotting, we observed significantly higher PTEN protein levels in thymic lymphomas derived from *Tp53^−^^/^^−^*; *Prl2^−^^/^^−^* mice compared with tumors derived from *Tp53^−^^/^^−^* mice (119% increase, *P* = 0.0014; Fig. 5A and B). *Prl2* deletion also resulted in significantly higher PTEN protein in sarcomas from the *Tp53-H* model (68.4% increase, *P* = 0.0434; Supplementary Fig. S4A and S4B). Accordingly, the increased PTEN protein level observed in tumors upon *Prl2* deletion was coupled with suppressed Akt activation in both the inducible *Tp53^−^^/^^−^* background (57.8% reduction, *P* = 0.0015; Fig. 5A and B) and the *Tp53-H* background by both Western blot and IHC staining (51.8% reduction, *P* = 0.0086; Supplementary Fig. S4A–S4D).

Akt activation regulates several downstream signaling processes through various Akt substrates that can affect tumor cell proliferation. We then performed further analysis to corroborate the reduced Akt activation we observed upon *Prl2* deletion with reduced Akt substrate phosphorylation that would support reduced proliferation. Cell growth and protein synthesis are cellular processes regulated by mTOR, which can be activated by Akt-mediated phosphorylation (33). As expected, we observed a significant reduction in mTORC1 phosphorylation in *Tp53^−^^/^^−^*; *Prl2^−^^/^^−^* mice, which is consistent with lower Akt activation (39% reduction, *P* = 0.0151; Fig. 5A and C). In addition, Akt inhibits glycogen synthase kinase 3β (GSK3β) through S9 phosphorylation which prevents accessibility to GSK3β substrates to promote cell proliferation and metabolism (33). The PRL2-deficient thymic lymphomas had reduced pS9 in GSK3β, which supports reduced proliferation observed in tumors derived from these mice (61% reduction, *P* = 0.0271; Fig. 5A and C). The ERK signaling pathway is known to regulate tumor growth and PRL2 has been shown to modulate this pathway *in vitro* (2, 8, 34), but not *in vivo* (9). We therefore assessed ERK activation, and no significant difference was observed between experimental and control groups (Supplementary Fig. S5A and S5B). Sarcomas derived from *Tp53-H*; *Prl2-KO* mice also showed reduced mTORC1 and GSK3β phosphorylation compared with *Tp53-het*–derived tumors, in accordance with reduced tumor proliferation observed (29.2% reduction, *P* = 0.0473, and 44.7% reduction, *P* = 0.0465, respectively Supplementary Fig. S4A–S4C).

The tumor suppressor P53 is a major regulator of cell apoptosis and we previously showed that *Prl2* deletion in *Pten* heterozygous mice promotes P53-dependent apoptosis. In our models of *Tp53* deficiency–induced tumorigenesis, we did not observe any difference in apoptosis upon *Prl2* deletion. Nonetheless, we assessed the protein expression level of some P53-regulated genes in thymic lymphomas derived from *Tp53^−^^/^^−^*; *Prl2^−^^/^^−^* and *Tp53^−^^/^^−^* mice, such as puma, BAX, p21. Our results confirm that *Prl2* deletion in this model does not affect the expression of these proteins, hence apoptosis is not affected upon *Prl2* deletion in this model (Fig. 5A and D). The forkhead box O (FoxO) proteins are transcription factors that regulate several cellular processes such as apoptosis, cell cycle, and stress response. FoxOs are direct Akt substrates, and their phosphorylation by Akt enables 14-3-3 binding to FOXO (33). This interaction prevents nuclear localization of FoxO, and thus inhibits its activity as a transcription factor. Thymic lymphomas from *Tp53^−^^/^^−^*; *Prl2^−^^/^^−^* mice had significantly lower FoxO-1 and -3 phosphorylation compared with the control mice (73.6% reduction, *P* = 00014, and 86.1% reduction, *P* = 0034; Fig. 5A and E). We showed that there are no differences in apoptosis and apoptotic signaling that could also be regulated by FoxOs upon *Prl2* deletion in this model. In addition to apoptosis, FoxOs also regulate genes involved in the cell cycle, and this regulatory function could impact cell proliferation. P27 and cyclin D1 are target genes for FoxO that are known to regulate cell-cycle progression. No changes in cyclin D1 were observed, but we saw a significant increase in p27 protein levels in *Tp53^−^^/^^−^*; *Prl2^−^^/^^−^* mice compared with *Tp53^−^^/^^−^* mice (113.1% increase, *P* = 0.0136; Fig. 5A and E). P27 promotes cell-cycle arrest, and this would be consistent with reduced proliferation observed in thymic lymphomas upon *Prl2* deletion. Taken together, our results show that loss of PRL2 in thymic lymphomas derived from *Tp53^−^^/^^−^* mice leads to PTEN augmentation and attenuation of Akt and downstream signaling. This inhibition of Akt signaling results in reduced tumor cell proliferation and reduced tumor burden in the mice, while the apoptotic pathway remains unaffected.

### Pharmacological Inhibition of PRL2 in Tamoxifen-inducible Tp53 Deletion Model Significantly Inhibits Thymic Lymphoma Growth

**FIGURE 6:**
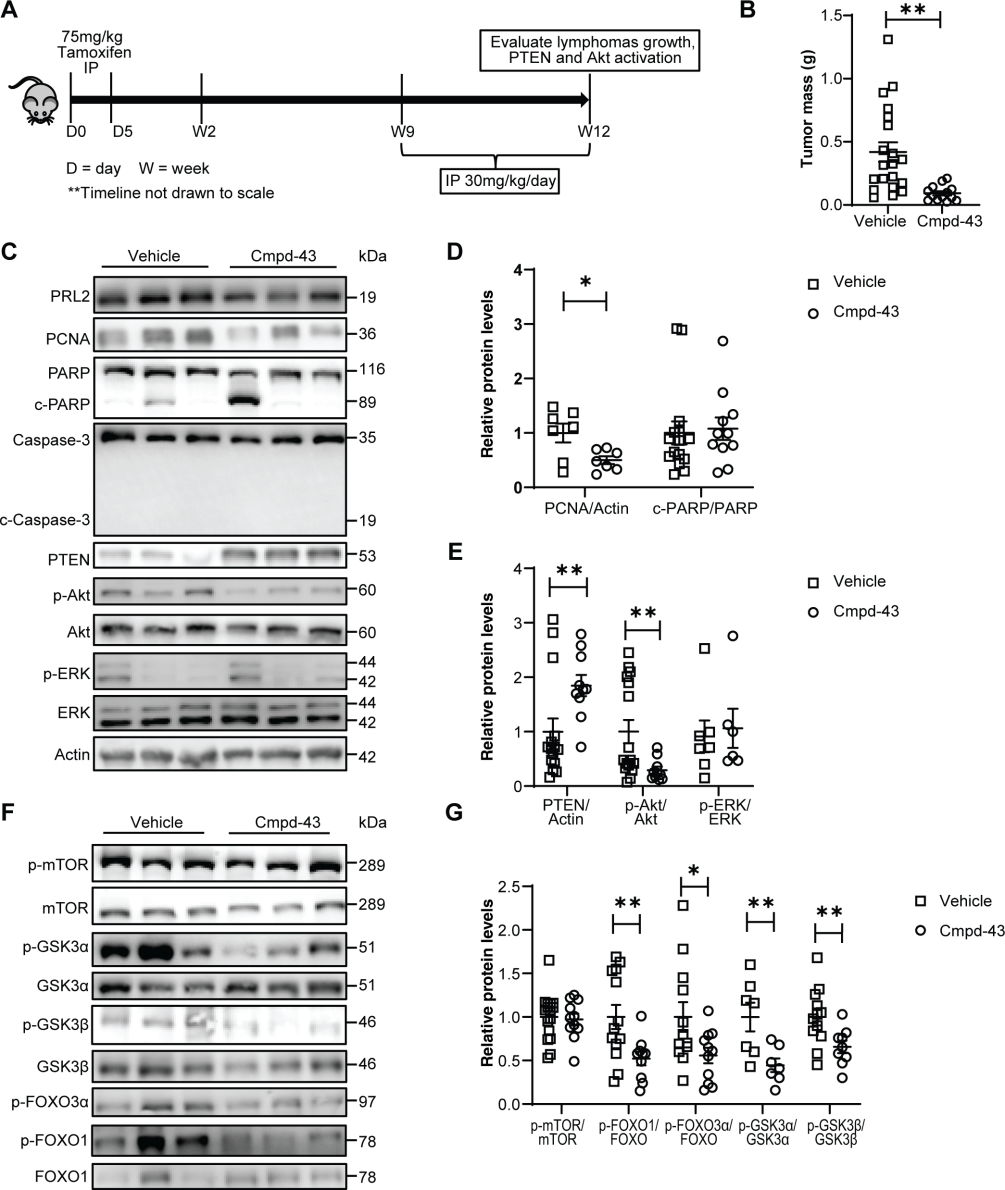
Pharmacologic inhibition of PRL2 in tamoxifen-inducible *Tp53^−^^/^^−^* mouse inhibits tumor proliferation through PTEN augmentation to hinder tumor growth. **A,** Experimental design for the treatment of inducible *Tp53^−^^/^^−^* with compound 43. **B,** Dot plot for mass of thymic lymphoma derived from inducible *Tp53^−^^/^^−^* treated with or without compound 43, data are shown as mean ± SEM, *Tp53^−^^/^^−^* + vehicle *n* = 20, *Tp53^−^^/^^−^* + compound 43 = 13. **C,** Representative Western blot analysis from thymic lymphomas derived from inducible *Tp53^−^^/^^−^* mice treated with vehicle or compound 43 to assess the levels PCNA, c-PARP, c-caspase 3, PTEN, and the activation of Akt and ERK1/2. **D,** Dot plot for quantification of C. **E,** Representative western blot from thymic lymphomas derived from inducible *Tp53^−^^/^^−^* mice treated with vehicle or compound 43 to assess the activation of Akt targets. **F,** Dot plot for quantification of E. Data are shown as mean ± SEM for samples obtained 12 weeks after tamoxifen treatment, *Tp53^−^^/^^−^* + vehicle *n* = 7–15, *Tp53^−^^/^^−^* + compound 43 *n* = 7–11; *, *P* < 0.05; **, *P* < 0.01.

Through a genetic approach, we successfully showed that deletion of *Prl2* inhibits thymic lymphoma growth in *Tp53^−^^/^^−^* background mice. In Figure 3D, we show that the thymus mass is similar between both experimental and control groups 9 weeks after tamoxifen treatment, but the tumors are significantly smaller in *Tp53^−^^/^^−^*; *Prl2^−^^/^^−^* mice at 12 weeks after tamoxifen. Tumor growth is significant between week 9 and week 12 for the *Tp53^−^^/^^−^* mice, and this provides an adequate window to assess pharmacologic inhibition of PRL2. We previously reported Cmpd-43 as a PRL inhibitor that disrupts PRL trimerization, which is essential for PRL function (24). Cmpd-43 efficiently inhibits tumor growth in a xenograft model (3, 24) as well as proliferation of leukemia cell lines *in vitro* and *in vivo* (35–37). To evaluate the therapeutic potential of PRL2 inhibition in *Tp53*-deficient models, we therefore assessed the ability of Cmpd-43 to suppress tumorigenesis in our inducible *Tp53^−^^/^^−^* mouse model. This will be the first time this compound is used in a genetically engineered cancer model. Thymic lymphoma development was induced as previously described in *Tp53^−^^/^^−^* mice, and 9 weeks after tamoxifen treatment, the mice were treated with either 30 mg/kg Cmpd-43 or vehicle control daily for 3 weeks, after which the mice were euthanized, and the tumors were analyzed (Fig. 6A). Consistent with previous reports using compound 43 *in vivo* with similar or longer dosage regiment than in our study presented here, we did not observe toxicity because of compound 43 treatment (24, 37). Mice treated with Cmpd-43 showed a significantly lower tumor burden, highlighting the efficacy of our PRL2 inhibitor to prevent tumorigenesis in this model (0.4189 g vs. 0.0929 g, *P* = 0.0020; Fig. 6B). Immunoblotting of the tumors derived from compound and vehicle-treated mice with the proliferation marker (PCNA) and apoptosis markers (PARP and caspase-3) showed a 50% reduction in tumor cell proliferation upon compound treatment, with no change in apoptosis (Fig. 6C and D). These results are consistent with our observations for the genetic inhibition of PRL2 in this model. We showed that genetic deletion of *Prl2* leads to PTEN augmentation and suppression of Akt signaling in this *Tp53^−^^/^^−^* model. We therefore immunoblotted for PTEN and Akt phosphorylation to determine whether pharmacologic inhibition of PRL2 with Cmpd-43 also elevates PTEN protein and inhibits Akt signaling to prevent tumorigenesis as observed. We observed approximately 85% increase in PTEN, and approximately 70% reduction in Akt phosphorylation following Cmpd-43 treatment (Fig. 6C and E). Reduced Akt activation was accompanied by reduced phosphorylation of downstream targets such as FoxOs (∼45% reduction), GSK3α (∼55% reduction), and GSK3β (∼35% reduction), which corroborates our observations in the genetic model (Fig. 6F and G). Interestingly, we did not observe any changes in mTOR phosphorylation. Reduced phosphorylation of FoxOs and GSK3s affects the expression of P27 (∼110% increase) and cyclin D1 (∼46% reduction) after pharmacologic inhibition of PRL2 (Supplementary Fig. S6A and S6B). These changes in Akt target phosphorylation as well as p27 and cyclin D1 expression support the smaller tumor size observed after Cmpd-43 treatment. These results suggest that trimer disruption with Cmpd-43 leads to PTEN augmentation and Akt inhibition in our inducible *Tp53^−^^/^^−^* tumor model. In addition, Cmpd-43 was previously shown to inhibit ERK signaling in a melanoma xenograft model and in cell culture. Just like in the genetic model, pharmacologic inhibition of PRL2 with Cmpd-43 did not affect ERK activation (Fig. 6C and E). In addition, no changes in the expression of the P53 targets p21, BAX and puma were observed upon treatment with Cmpd-43 (Supplementary Fig. S6A and S6B). Taken together, pharmacologic inhibition of PRL2 with Cmpd-43 in our inducible *Tp53^−^^/^^−^* model phenocopied the results observed with genetic deletion of *Prl2* in this model.

## Discussion

The PRL phosphatases have garnered interest as therapeutic targets for cancer treatment due to their overexpression and oncogenicity to drive malignancies that correlate with poor survival prognosis (38–41). We recently showed that deletion of *Prl2* in a *Pten* heterozygous background results in PTEN augmentation and impedes *Pten* deficiency–induced spontaneous tumorigenesis by inhibiting tumor cell proliferation and promoting their apoptosis (9). In that work, we showed that mechanistically, PRL2 dephosphorylates PTEN on Y336 to promote its ubiquitination by NEDD4 and subsequent degradation. The work we are reporting here shows that PTEN augmentation resulting from genetic or pharmacologic inhibition of PRL2 can be exploited therapeutically to prevent cancer development in cancers that develop independently from PTEN inactivation and PI3K/Akt signaling hyperactivation. On the basis of cBioPortal cancer genomic database, more than 90% of all cancers develop without PTEN alterations, which emphasizes the importance of proving the efficacy of PTEN augmentation therapy in such cancers. We used the well-established *Tp53* deficiency tumor models to test this and confirmed PTEN augmentation as a result of both *Prl2* genetic deletion and PRL inhibition in *Tp53*-deficient mice. The tumor profile we observed from both models was consistent with literature reports (23, 27, 42, 43).

We developed a model for conditional *Prl2* deletion to control the where and when PRL2 could be deleted in mice, which would allow us to better study the function(s) of PRL2 in specific cancer types and in normal physiology. In this work, we induced whole body deletion of both *Prl2* and *Tp53* in the mice at the same time. *Tp53*-null mice predominantly developed thymic lymphomas and *Prl2* deletion in this model significantly delayed the development of these lymphomas. This model allowed us to primarily study the effect of *Prl2* deletion on *Tp53*-null thymic lymphomas, which accumulate hallmark mutations observed in T-cell acute lymphoblastic leukemia in humans (21). Recent work showed that deletion of Akt1 in *Tp53^−^^/^^−^* mice prevents the development of thymic lymphomas (30), and thus we would expect that inhibition of PRL2 to increase PTEN protein level and attenuate Akt signaling in such a model would also hinder tumor growth. The deletion of Akt1 in *Tp53^−^^/^^−^* mice resulted in approximately 85% increase in survival time (30), compared with approximately 30% increase in the work we present here. As expected, we showed that *Prl2* deletion prevents tumorigenesis in this model through PTEN augmentation and Akt inhibition. Unlike the model that used *Akt*-null mice, and thus completely prevented all Akt signaling, we only have partial inhibition of Akt signaling upon *Prl2* deletion. Thus, such differences in the effect of *Prl2* deletion compared with *Akt* deletion can be expected. Although the inhibition of PI3K/Akt pathway component appears promising for cancer treatment based on laboratory research, the development of approved medication that directly target members of this signaling pathway has been challenging because of on-target and off-target toxicities associated with direct Akt inhibition (44, 45). As an alternative approach, an increase in PTEN level is expected to oppose PI3K/Akt signaling activation and may have therapeutic benefits against cancer. Indeed, PTEN overexpression in mice has little adverse effect and has been shown to confer a tumor resistant phenotype (46, 47). Also, constitutive *Prl2^−^^/^^−^* mice have a normal life span even though their PTEN levels were 25% higher than the WT controls, and these mice show no other physiologic defect besides reduced body weight compared with control, which is consistent with observations in PTEN-overexpressing mice (7, 9). Consequently, PTEN restoration or augmentation therapy is a viable approach for cancer treatment that could circumvent the challenges faced by the inhibition of other members of the PI3K/Akt signaling pathway (48–50). In addition, PTEN has been shown to have anticancer function that is independent of its canonical role in the PI3K/Akt signaling pathway (12, 48, 50). Consequently, inhibition of PRL2 for PTEN augmentation would provide a viable anticancer approach, and the work presented by our group strongly supports the anticancer benefits of this approach.

We previously showed that Cmpd-43 (24), a small-molecule PRL detrimerizer, can prevent tumorigenesis or leukemogenesis in cancer cell-derived xenograft models and patient-derived xenograft models (24, 36, 37). For the first time here, we assessed the efficacy of these compounds in suppressing tumorigenesis *in vivo* in a genetically modified mouse cancer model. Because we had determined the growth kinetics of thymic lymphoma in *Tp53^−^^/^^−^* mice, we used this model to assess the efficacy of our compound. We observed significant tumor growth inhibition after 3 weeks of treatment with Cmpd-43, and just like we saw with genetic deletion of *Prl2*, we found that pharmacologic inhibition of PRL2 suppresses Akt signaling through PTEN augmentation to inhibit tumor cell proliferation with no effect on apoptosis in the thymic lymphomas. Also, although both Akt and ERK attenuation has been previously reported upon PRL downregulation *in vitro*, the effect on ERK has not been observed in *in vivo* models. Interestingly, treatment of these mice with Cmpd-43 over 3 weeks for PRL2 inhibition had comparable effect on tumor size as the genetic deletion of PRL2 which occurred at the same time as *Tp53* deletion, with no obvious adverse effect as also noted previously (24, 37). These results are much better than one would expect, and this is potentially due to Cmpd-43 being able to inhibit PRL1 and PRL3 in addition to PRL2 to provide synergistic effect on inhibition of tumor growth (24). Although we did not observe any improvement in survival when *Prl1* or *Prl3* were independently deleted in *Tp53*-null mice background (Supplementary Fig. S7), it is possible that combined inhibition of PRL1 and PRL2 could synergistically affect tumorigenesis. We previously observed such synergistic effect upon deletion of both PRL1 and PRL2 to impair spermatogenesis, while PRL1 deletion alone had no effect on spermatogenesis (6). Thus, inhibition of multiple PRLs by pan-PRL inhibitors such as Cmpd-43 in cancer cells could be more effective compared with single PRL inhibition. An additional advantage of Cmpd-43 is that it exploits an oligomerization mechanism unique to PRL therefore ensuring high selectivity over other PTPs. However, future studies are needed to develop PRL isoform-specific detrimerizers or inhibitors to further assess the therapeutic effectiveness of targeting a single PRL protein.

Interestingly, previous *in vitro* studies showed that PRL1 and PRL3 can downregulate p53 expression, and the expression of these PRLs is also transcriptionally regulated by p53 conversely (51–53). However, no such observation was made for PRL2. We previously found that *Prl2* deletion stabilized p53 though PI3K-Akt-MDM2-p53 axis, which promoted tumor cell apoptosis in lymphomas induced by PTEN deficiency (9). In the current study with *Tp53* deficiency tumor models, we did not observe any impact on the expression of p53 targets or apoptosis upon *Prl2* deletion in sarcoma or thymic lymphoma from both *Tp53*-H and *Tp53*-null tumors. These data suggest that the impact of *Prl2* on p53 is tissue/tumor type specific. More studies are required to further assess the underlying mechanism of whether or how PRL2 controls p53 under different conditions.

The tumor suppressor P53 is the most mutated and inactivated tumor suppressor in human cancers, and its dysregulation promotes a more severe cancer phenotype. Current therapeutic approaches under development for the treatment of such cancers involve inhibition of p53 degradation for cancers expressing WT p53 with or without lower protein expression and restoration of WT p53 function in cancers with mutated or truncated p53 expression (18, 54). Unfortunately, none of these approaches have been approved for clinical use due to p53-dependent and independent toxicity (54). This highlights the need for novel therapies that target cancers with p53 mutation or downregulation, and through this work we provide the proof-of-concept for PTEN augmentation approach via PRL2 inhibition. Previous work on PRL2, and the work we report here, show that loss of PRL2 expression or PRL2 inhibition has no adverse effect on survival, but instead promotes an antitumor phenotype through PTEN augmentation. Up to 80% of patients with sarcoma have P53 mutation or downregulation, but almost no PTEN mutation, which makes these tumors good targets for PTEN augmentation therapy. In mouse models of *Tp53* deficiency, genetic loss of *Prl2* or pharmacologic inhibition of PRL2 led to PTEN augmentation and attenuation of Akt signaling, which ultimately inhibited tumor cell proliferation to prevent tumor growth and promote survival in these tumor models. In conclusion, through this work, we not only further established the mechanistic regulation of PTEN by PRL2 but also demonstrated that genetic and pharmacologic inhibition of PRL2 *in vivo* could be exploited for PTEN augmentation therapy for the treatment of *Tp53*-deficient tumors.

## Supplementary Material

Figure S1 Representative flowchart for flow cytometry analysis

Figure S2 Experiemental design for tamoxifen inducible Tp53 deletion w/o Prl2 deletion

Figure S3 Prl2 deletion inhibits tumor cell proliferations

Figure S4 Prl2 deletion upregulates PTEN to inhibit Akt signaling and hinder tumor cell proliferation in Tp53 heterozygous mice

Figure S5 Prl2 deletion does not affect Erk activation in inducible Tp53-/- mice derived thymic lymphomas

Figure S6 Effect of pharmacological inhibition of PRL2 in inducible Tp53-/- model on P53 and FoxO targets

Figure S7 Loss of PRL1 or PRL3 does not improve tumor-free survival of Tp53-null mice

## References

[1] Bessette DC , Qiu D, Pallen CJ. PRL PTPs: mediators and markers of cancer progression. Cancer Metastasis Rev 2008;27:231–52. 18224294 10.1007/s10555-008-9121-3

[2] Hardy S , Wong NN, Muller WJ, Park M, Tremblay ML. Overexpression of the protein tyrosine phosphatase PRL-2 correlates with breast tumor formation and progression. Cancer Res 2010;70:8959–67. 20841483 10.1158/0008-5472.CAN-10-2041

[3] Frankson R , Yu ZH, Bai Y, Li Q, Zhang RY, Zhang ZY. Therapeutic targeting of oncogenic tyrosine phosphatases. Cancer Res 2017;77:5701–5. 28855209 10.1158/0008-5472.CAN-17-1510 PMC5827927

[4] Hardy S , Kostantin E, Hatzihristidis T, Zolotarov Y, Uetani N, Tremblay ML. Physiological and oncogenic roles of the PRL phosphatases. FEBS J 2018;285:3886–908. 29770564 10.1111/febs.14503

[5] Dong Y , Zhang L, Bai Y, Zhou HM, Campbell AM, Chen H, . Phosphatase of regenerating liver 2 (PRL2) deficiency impairs kit signaling and spermatogenesis. J Biol Chem 2014;289:3799–810. 24371141 10.1074/jbc.M113.512079 PMC3916576

[6] Bai Y , Zhou HM, Zhang L, Dong Y, Zeng Q, Shou W, . Role of phosphatase of regenerating liver 1 (PRL1) in spermatogenesis. Sci Rep 2016;6:34211. 27666520 10.1038/srep34211 PMC5035919

[7] Dong Y , Zhang L, Zhang S, Bai Y, Chen H, Sun X, . Phosphatase of regenerating liver 2 (PRL2) is essential for placental development by down-regulating PTEN (phosphatase and tensin homologue deleted on chromosome 10) and activating Akt protein. J Biol Chem 2012;287:32172–9. 22791713 10.1074/jbc.M112.393462 PMC3442547

[8] Kobayashi M , Nabinger SC, Bai Y, Yoshimoto M, Gao R, Chen S, . Protein tyrosine phosphatase PRL2 mediates notch and kit signals in early T cell progenitors. Stem Cells 2017;35:1053–64. 28009085 10.1002/stem.2559 PMC5367971

[9] Li Q , Bai Y, Lyle LT, Yu G, Amarasinghe O, Meke FN, . Mechanism of PRL2 phosphatase-mediated PTEN degradation and tumorigenesis. Proc Natl Acad Sci U S A 2020;117:20538–48. 32788364 10.1073/pnas.2002964117 PMC7456095

[10] Salmena L , Carracedo A, Pandolfi PP. Tenets of PTEN tumor suppression. Cell 2008;133:403–14. 18455982 10.1016/j.cell.2008.04.013

[11] Alimonti A , Carracedo A, Clohessy JG, Trotman LC, Nardella C, Egia A, . Subtle variations in Pten dose determine cancer susceptibility. Nat Genet 2010;42:454–8. 20400965 10.1038/ng.556 PMC3118559

[12] Milella M , Falcone I, Conciatori F, Incani UC, Del Curatolo A, Inzerilli N, . PTEN: multiple functions in human malignant tumors. Front Oncol 2015;5:24. 25763354 10.3389/fonc.2015.00024 PMC4329810

[13] Bernstein C , Prasad A, Nfonsam V, Bernstein H. DNA damage, DNA repair and cancer. In: Chen C, editor. New research directions in DNA repair. London: IntechOpen; 2013. p. 413–47.

[14] Olivier M , Hussain SP, Caron de Fromentel C, Hainaut P, Harris CC. TP53 mutation spectra and load: a tool for generating hypotheses on the etiology of cancer. IARC Sci Publ 2004;247–70. 15055300

[15] Vogelstein B , Lane D, Levine AJ. Surfing p53 network. Nature 2000;408:307–10. 11099028 10.1038/35042675

[16] Thoenen E , Curl A, Iwakuma T. TP53 in bone and soft tissue sarcomas. Pharmacol Ther 2019;202:149–64. 31276706 10.1016/j.pharmthera.2019.06.010 PMC6746598

[17] Wadayama B , Toguchida J, Yamaguchi T, Sasaki MS, Kotoura Y, Yamamuro T. P53 expression and its relationship to DNA alterations in bone and soft tissue sarcomas. Br J Cancer 1993;68:1134–9. 8260365 10.1038/bjc.1993.493 PMC1968651

[18] Hu J , Cao J, Topatana W, Juengpanich S, Li S, Zhang B, . Targeting mutant p53 for cancer therapy: direct and indirect strategies. J Hematol Oncol 2021;14:157. 34583722 10.1186/s13045-021-01169-0 PMC8480024

[19] Wang Z , Sun Y. Targeting p53 for novel anticancer therapy. Transl Oncol 2010;3:1–12. 20165689 10.1593/tlo.09250 PMC2822448

[20] Lane DP , Cheok CF, Lain S. p53-based cancer therapy. Cold Spring Harb Perspect Biol 2010;2:a001222. 20463003 10.1101/cshperspect.a001222 PMC2926755

[21] Dudgeon C , Chan C, Kang W, Sun Y, Emerson R, Robins H, . The evolution of thymic lymphomas in p53 knockout mice. Genes Dev 2014;28:2613–20. 25452272 10.1101/gad.252148.114 PMC4248292

[22] Hinkal G , Parikh N, Donehower LA. Timed somatic deletion of p53 in mice reveals age-associated differences in tumor progression. PLoS One 2009;4:e6654. 19680549 10.1371/journal.pone.0006654 PMC2721630

[23] Harvey M , Mcarthur MJ, Montgomery CA Jr, Butel JS, Bradley A, Donehower LA. Spontaneous and carcinogen-induced tumorigenesis in p53-deficient mice. Nat Genet 1993;5:225–9. 8275085 10.1038/ng1193-225

[24] Bai Y , Yu ZH, Liu S, Zhang L, Zhang RY, Zeng LF, . Novel anticancer agents based on targeting the trimer interface of the PRL phosphatase. Cancer Res 2016;76:4805–15. 27325652 10.1158/0008-5472.CAN-15-2323 PMC4987244

[25] Carlock C , Bai Y, Paige-Hood A, Li Q, Nguele Meke F, Zhang ZY. PRL2 inhibition elevates PTEN protein and ameliorates progression of acute myeloid leukemia. JCI Insight 2023;8:e170065. 37665633 10.1172/jci.insight.170065 PMC10619439

[26] Haines BB , Ryu CJ, Chang S, Protopopov A, Luch A, Kang YH, . Block of T cell development in P53-deficient mice accelerates development of lymphomas with characteristic RAG-dependent cytogenetic alterations. Cancer Cell 2006;9:109–20. 16473278 10.1016/j.ccr.2006.01.004

[27] Kemp CJ , Wheldon T, Balmain A. p53-deficient mice are extremely susceptible to radiation-induced tumorigenesis. Nat Genet 1994;8:66–9. 7987394 10.1038/ng0994-66

[28] Mao JH , Wu D, Perez-Losada J, Nagase H, DelRosario R, Balmain A. Genetic interactions between Pten and p53 in radiation-induced lymphoma development. Oncogene 2003;22:8379–85. 14627978 10.1038/sj.onc.1207083

[29] Marusyk A , Porter CC, Zaberezhnyy V, DeGregori J. Irradiation selects for p53-deficient hematopoietic progenitors. PLoS Biol 2010;8:e1000324. 20208998 10.1371/journal.pbio.1000324 PMC2830447

[30] Yu WN , Nogueira V, Sobhakumari A, Patra KC, Bhaskar PT, Hay N. Systemic Akt1 deletion after tumor onset in p53-/- mice increases lifespan and regresses thymic lymphoma emulating p53 restoration. Cell Rep 2015;12:610–21. 26190111 10.1016/j.celrep.2015.06.057 PMC4526157

[31] Trakala M , Aggarwal M, Sniffen C, Zasadil L, Carroll A, Ma D, . Clonal selection of stable aneuploidies in progenitor cells drives high-prevalence tumorigenesis. Genes Dev 2021;35:1079–92. 34266888 10.1101/gad.348341.121 PMC8336892

[32] Lee CL , Castle KD, Moding EJ, Blum JM, Williams N, Luo L, . Acute DNA damage activates the tumour suppressor p53 to promote radiation-induced lymphoma. Nat Commun 2015;6:8477. 26399548 10.1038/ncomms9477 PMC4586051

[33] Manning BD , Toker A. AKT/PKB signaling: navigating the network. Cell 2017;169:381–405. 28431241 10.1016/j.cell.2017.04.001 PMC5546324

[34] Wang Y , Lazo JS. Metastasis-associated phosphatase PRL-2 regulates tumor cell migration and invasion. Oncogene 2012;31:818–27. 21765462 10.1038/onc.2011.281 PMC4553949

[35] Kobayashi M , Chen S, Bai Y, Yao C, Gao R, Sun X-J, . Phosphatase PRL2 promotes AML1-ETO-induced acute myeloid leukemia. Leukemia 2017;31:1453–7. 28220038 10.1038/leu.2017.67 PMC5695226

[36] Kobayashi M , Bai Y, Chen S, Gao R, Yao C, Cai W, . Phosphatase PRL2 promotes oncogenic NOTCH1-induced T-cell leukemia. Leukemia 2017;31:751–4. 27872499 10.1038/leu.2016.340 PMC5695227

[37] Chen H , Bai Y, Kobayashi M, Xiao S, Cai W, Barajas S, . PRL2 phosphatase enhances oncogenic FLT3 signaling via dephosphorylation of the E3 ubiquitin ligase CBL at tyrosine 371. Blood 2023;141:244–59. 36206490 10.1182/blood.2022016580 PMC9936309

[38] Jin S , Wang K, Xu K, Xu J, Sun J, Chu Z, . Oncogenic function and prognostic significance of protein tyrosine phosphatase PRL-1 in hepatocellular carcinoma. Oncotarget 2014;5:3685–96. 25003523 10.18632/oncotarget.1986 PMC4116513

[39] Wang H , Quah SY, Jing MD, Manser E, Jing PT, Zeng Q. PRL-3 down-regulates PTEN expression and signals through PI3K to promote epithelial-mesenchymal transition. Cancer Res 2007;67:2922–6. 17409395 10.1158/0008-5472.CAN-06-3598

[40] Xing X , Peng L, Qu L, Ren T, Dong B, Su X, . Prognostic value of PRL-3 overexpression in early stages of colonic cancer. Histopathology 2009;54:309–18. 19236507 10.1111/j.1365-2559.2009.03226.x

[41] Bardelli A , Saha S, Sager JA, Romans KE, Xin B, Markowitz SD, . PRL-3 expression in metastatic cancers. Clin cancer Res 2003;9:5607–15. 14654542

[42] Landuzzi L , Ianzano ML, Nicoletti G, Palladini A, Grosso V, Ranieri D, . Genetic prevention of lymphoma in p53 knockout mice allows the early development of p53-related sarcomas. Oncotarget 2014;5:11924–38. 25426555 10.18632/oncotarget.2650 PMC4322986

[43] Donehower LA , Harvey M, Vogel H, McArthur MJ, Montgomery CA, Park SH, . Effects of genetic background on tumorigenesis in p53-deficient mice. Mol Carcinog 1995;14:16–22. 7546219 10.1002/mc.2940140105

[44] Martorana F , Motta G, Pavone G, Motta L, Stella S, Vitale SR, . AKT inhibitors: new weapons in the fight against breast cancer? Front Pharmacol 2021;12:662232. 33995085 10.3389/fphar.2021.662232 PMC8118639

[45] Janku F . Phosphoinositide 3-kinase (PI3K) pathway inhibitors in solid tumors: from laboratory to patients. Cancer Treat Rev 2017;59:93–101. 28779636 10.1016/j.ctrv.2017.07.005

[46] Dupont J , Renou JP, Shani M, Hennighausen L, LeRoith D. PTEN overexpression suppresses proliferation and differentiation and enhances apoptosis of the mouse mammary epithelium. J Clin Invest 2002;110:815–25. 12235113 10.1172/JCI13829 PMC151121

[47] Garcia-Cao I , Song MS, Hobbs RM, Laurent G, Giorgi C, De Boer VCJ, . Systemic elevation of PTEN induces a tumor-suppressive metabolic state. Cell 2012;149:49–62. 22401813 10.1016/j.cell.2012.02.030 PMC3319228

[48] Hopkins BD , Parsons RE. Molecular pathways: Intercellular PTEN and the potential of PTEN restoration therapy. Clin Cancer Res 2014;20:5379–83. 25361917 10.1158/1078-0432.CCR-13-2661 PMC4362520

[49] Islam MA , Xu Y, Zope H, Cao W, Mahmoudi M, Langer R, . Restoration of tumor suppression *in vivo* by systemic delivery of chemically-modified PTEN mRNA nanoparticles. J Clin Oncol 35:15s, 2017 (suppl; asbtr 11582).

[50] Lee YR , Chen M, Pandolfi PP. The functions and regulation of the PTEN tumour suppressor: new modes and prospects. Nat Rev Mol Cell Biol 2018;19:547–62. 29858604 10.1038/s41580-018-0015-0

[51] Min SH , Kim DM, Heo YS, Kim YI, Kim HM, Kim J, . New p53 target, phosphatase of regenerating liver 1 (PRL-1) downregulates p53. Oncogene 2009;28:545–54. 18997816 10.1038/onc.2008.409

[52] Min SH , Kim DM, Heo YS, Kim HM, Kim IC, Yoo OJ. Downregulation of p53 by phosphatase of regenerating liver 3 is mediated by MDM2 and PIRH2. Life Sci 2010;86:66–72. 19945467 10.1016/j.lfs.2009.11.010

[53] Basak S , Jacobs SB, Krieg AJ, Pathak N, Zeng Q, Kaldis P, . The metastasis-associated gene Prl-3 is a p53 target involved in cell-cycle regulation. Mol Cell 2008;30:303–14. 18471976 10.1016/j.molcel.2008.04.002 PMC3951836

[54] Hassin O , Oren M. Drugging p53 in cancer: one protein, many targets. Nat Rev Drug Discov 2023;22:127–44. 36216888 10.1038/s41573-022-00571-8 PMC9549847

