# Supplementary material for: Inhibition of PRL2 Upregulates PTEN and Attenuates Tumor Growth in Tp53-deficient Sarcoma and Lymphoma Mouse Models
Source: Cancer Res Commun. 2024 Jan 2;4(1):5–17. doi: 10.1158/2767-9764.CRC-23-0308 (PMC10764713; doi:10.1158/2767-9764.CRC-23-0308)
Supplement: Figure S1 — Representative flowchart for flow cytometry analysis [file crc-23-0308-s01.pdf]

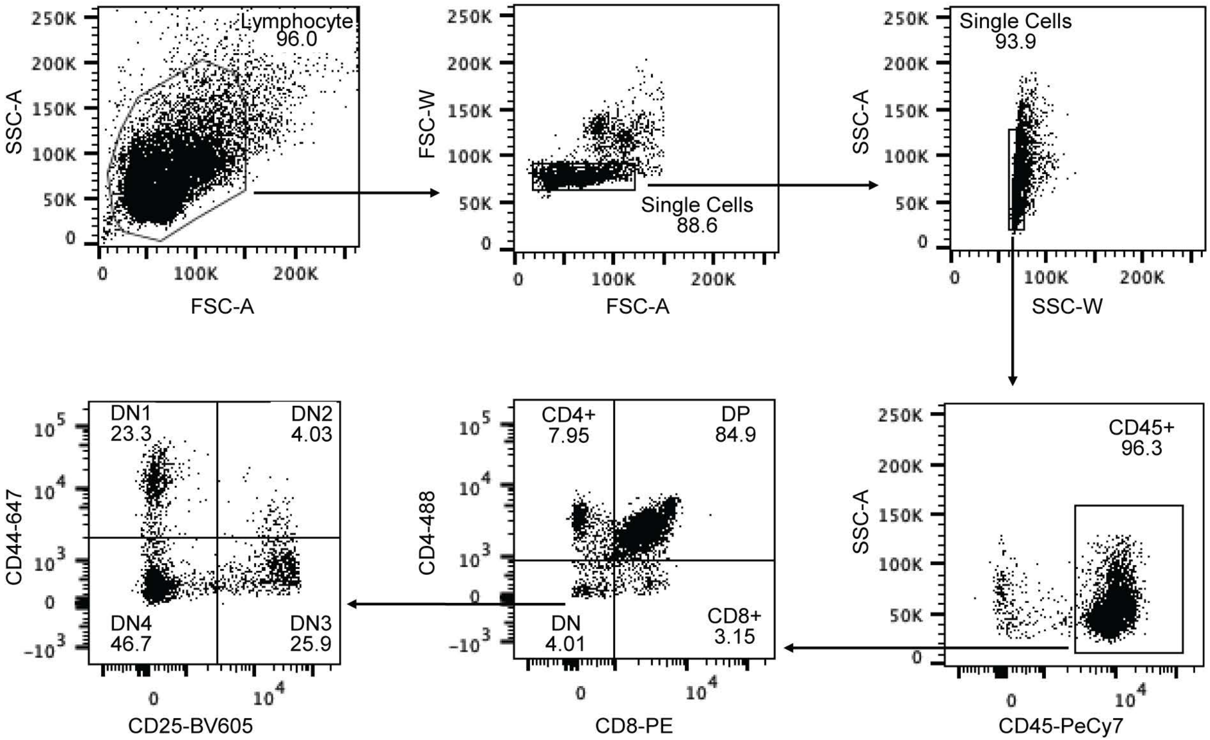

Supplementary Figure 1. Representative flow chart for flow cytometry analysis. Total cells analyzed from thymus (normal thymus or thymic lymphomas) were gated using different combinations of forward and side scatter plots to select single cell population. Single cells were then gated with CD45 to select the CD45+ lymphocytes. These were then gated using CD4 and CD8 markers, and the following populations were denoted: CD4+CD8+ double positive (DP), CD4+ cells, CD8+ cells, and CD4- CD8- double negative (DN) populations. The DN population was then selected and gated using CD44 and CD25 markers to obtain the following populations: DN1, CD44+CD25- ; DN2, CD44+CD25+; DN3, CD44-CD25+; DN4, CD44-CD25-. This gating strategy was used to obtain population ratios shown in figure 3E and 3F.
