# Supplementary material for: Inhibition of PRL2 Upregulates PTEN and Attenuates Tumor Growth in Tp53-deficient Sarcoma and Lymphoma Mouse Models
Source: Cancer Res Commun. 2024 Jan 2;4(1):5–17. doi: 10.1158/2767-9764.CRC-23-0308 (PMC10764713; doi:10.1158/2767-9764.CRC-23-0308)
Supplement: Figure S2 — Experiemental design for tamoxifen inducible Tp53 deletion w/o Prl2 deletion [file crc-23-0308-s02.pdf]

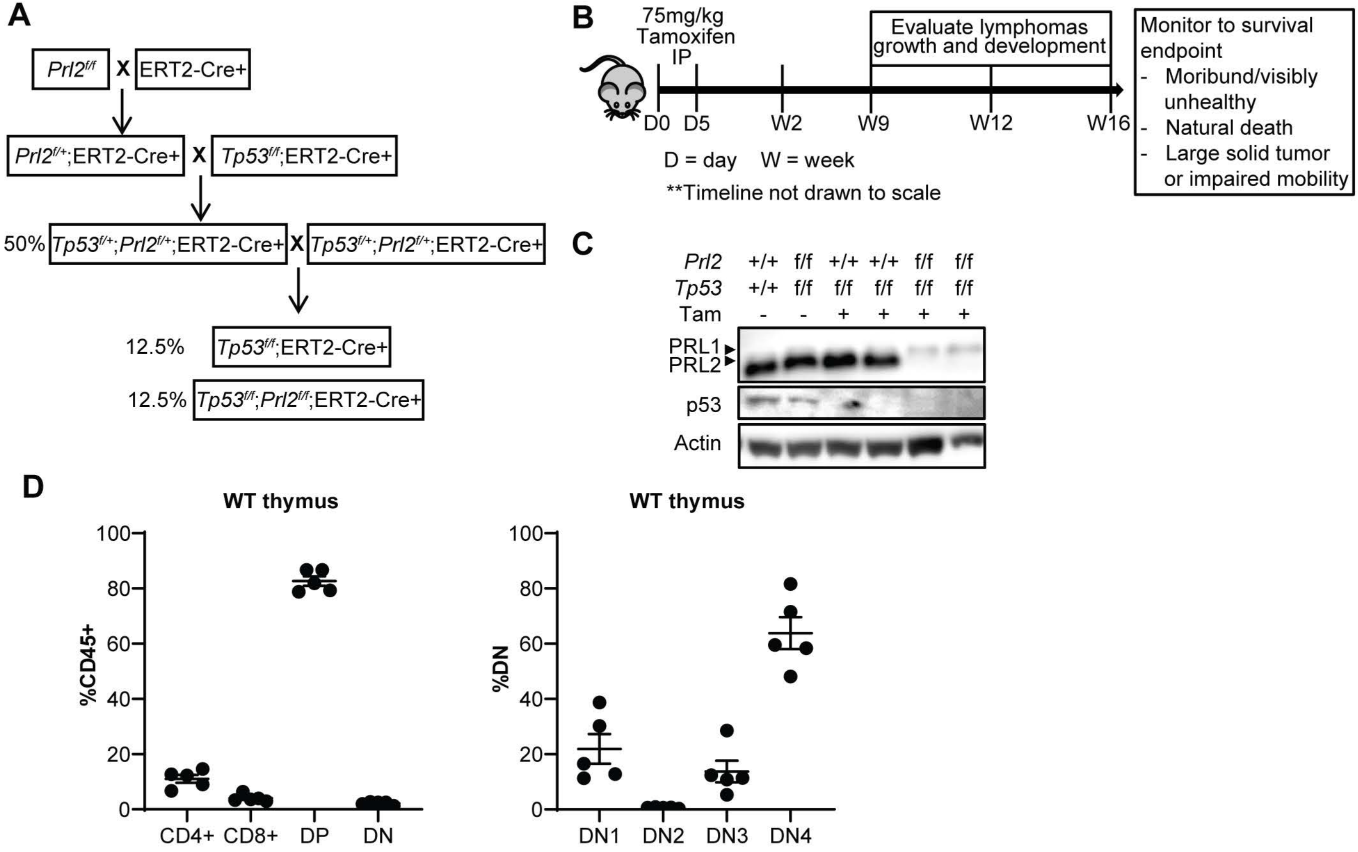

Supplementary Figure 2. Experimental design for tamoxifen inducible *Tp53* deletion w/o *Prl2* deletion. A) Breeding scheme to obtain inducible control *Tp53*$^{f/f}$ ERT2-Cre$^{+/+}$ and inducible experimental *Tp53*$^{f/f}$ *Prl2*$^{f/f}$ ERT2-Cre$^{+/+}$ mice. B) Experimental design and timeline for the treatment of tamoxifen and isolation of thymic lymphomas. C) Representative western blot to confirm the deletion of *Prl2* and/or *Tp53* after tamoxifen treatment. D) Thymocyte population ratios in wild type mice thymus. DP: Double Positive (CD4+ CD8+). DN: Double Negative (CD4- CD8-).
