# Supplementary material for: Inhibition of PRL2 Upregulates PTEN and Attenuates Tumor Growth in Tp53-deficient Sarcoma and Lymphoma Mouse Models
Source: Cancer Res Commun. 2024 Jan 2;4(1):5–17. doi: 10.1158/2767-9764.CRC-23-0308 (PMC10764713; doi:10.1158/2767-9764.CRC-23-0308)
Supplement: Figure S3 — Prl2 deletion inhibits tumor cell proliferations [file crc-23-0308-s03.pdf]

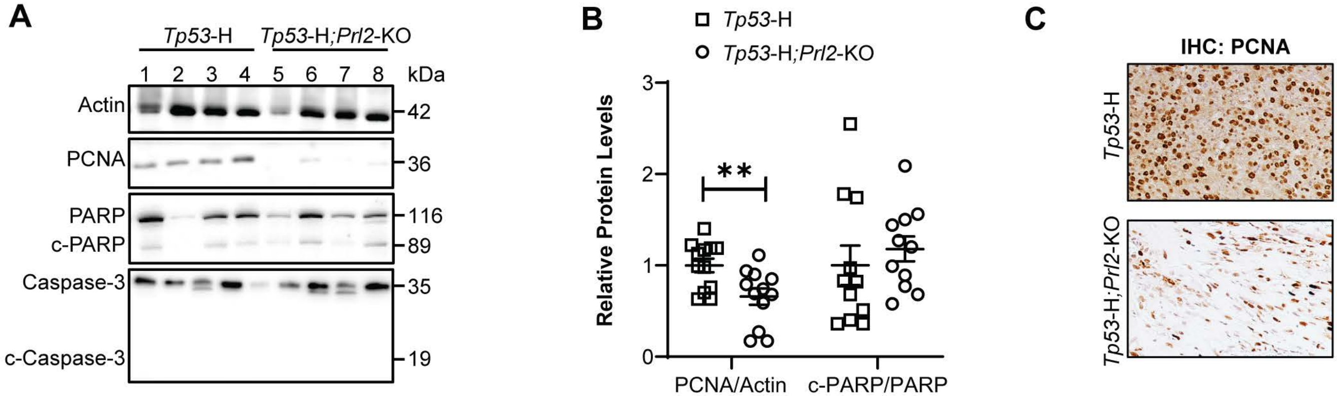

Supplementary Figure 3. *Prl2* deletion inhibits tumor cell proliferation but not apoptosis in *Tp53* heterozygous mice. A) Representative western blot from sarcomas derived from *Tp53-H* and *Tp53-H Prl2-KO* mice to determine the expression of proliferation marker PCNA and apoptosis markers c-PARP and c-caspase 3. B) Quantification of proliferation and apoptosis markers relative to actin and total protein control respectively for western blots in (A), error bars represent the SEM, *Tp53-H* n = 12, *Tp53-H Prl2-KO* n = 11. C) Representative IHC stain using PCNA marker to compare proliferation in sarcomas derived from *Tp53-H* and *Tp53-H Prl2-KO* mice. ** $p<0.01$
