# Supplementary material for: Inhibition of PRL2 Upregulates PTEN and Attenuates Tumor Growth in Tp53-deficient Sarcoma and Lymphoma Mouse Models
Source: Cancer Res Commun. 2024 Jan 2;4(1):5–17. doi: 10.1158/2767-9764.CRC-23-0308 (PMC10764713; doi:10.1158/2767-9764.CRC-23-0308)
Supplement: Figure S4 — Prl2 deletion upregulates PTEN to inhibit Akt signaling and hinder tumor cell proliferation in Tp53 heterozygous mice [file crc-23-0308-s04.pdf]

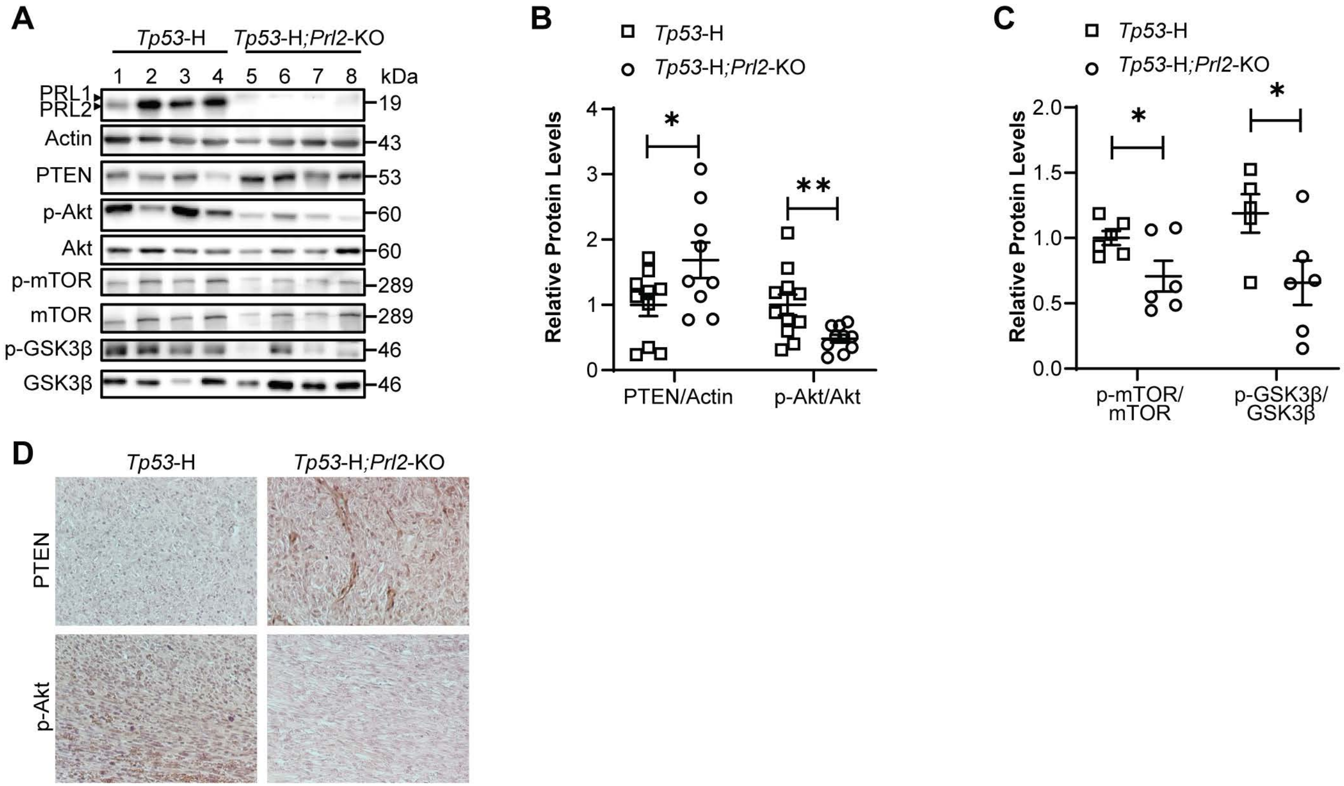

Supplementary Figure 4. *Prl2* deletion upregulates PTEN to inhibit Akt signaling and hinder tumor cell proliferation in *Tp53* heterozygous mice. A) Representative western blot from *Tp53-H* and *Tp53-H Prl2-KO* derived sarcomas to determine expression of PTEN, Akt and downstream targets of Akt. B) Quantification for PTEN and Akt immunoblots shown in (A), error bars represent the SEM, *Tp53-H* n = 10, *Tp53-H Prl2-KO* n = 10. C) Quantification for mTOR and GSK3β immunoblots shown in (A), error bars represent the SEM, *Tp53-H* n = 6, *Tp53-H Prl2-KO* n = 6. D) Representative IHC stain for PTEN and Akt expression in sarcomas derived by *Tp53-H* and *Tp53-H Prl2-KO* mice. * $p<0.05$, ** $p<0.01$.
