# Supplementary material for: Inhibition of PRL2 Upregulates PTEN and Attenuates Tumor Growth in Tp53-deficient Sarcoma and Lymphoma Mouse Models
Source: Cancer Res Commun. 2024 Jan 2;4(1):5–17. doi: 10.1158/2767-9764.CRC-23-0308 (PMC10764713; doi:10.1158/2767-9764.CRC-23-0308)
Supplement: Figure S5 — Prl2 deletion does not affect Erk activation in inducible Tp53-/- mice derived thymic lymphomas [file crc-23-0308-s05.pdf]

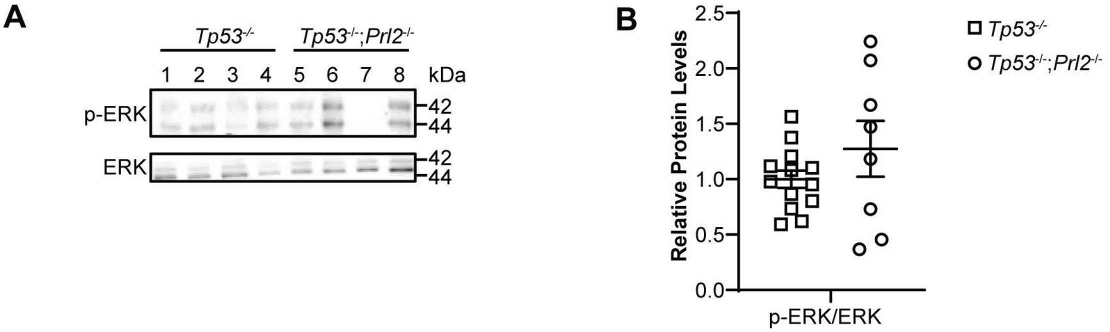

Supplementary Figure 5. *Prl2* deletion does not affect Erk activation in inducible *Tp53*$^{-/-}$ mice derived thymic lymphomas. A) Representative western blot from inducible *Tp53*$^{-/-}$ and inducible *Tp53*$^{-/-}$ *Prl2*$^{-/-}$ derived thymic lymphomas to determine ERK activation. B) Quantification for (A), error bars represent the SEM, *Tp53*$^{-/-}$ n = 13, *Tp53*$^{-/-}$ *Prl2*$^{-/-}$n = 8.
