# Supplementary material for: Inhibition of PRL2 Upregulates PTEN and Attenuates Tumor Growth in Tp53-deficient Sarcoma and Lymphoma Mouse Models
Source: Cancer Res Commun. 2024 Jan 2;4(1):5–17. doi: 10.1158/2767-9764.CRC-23-0308 (PMC10764713; doi:10.1158/2767-9764.CRC-23-0308)
Supplement: Figure S6 — Effect of pharmacological inhibition of PRL2 in inducible Tp53-/- model on P53 and FoxO targets [file crc-23-0308-s06.pdf]

Supplementary Figure 6

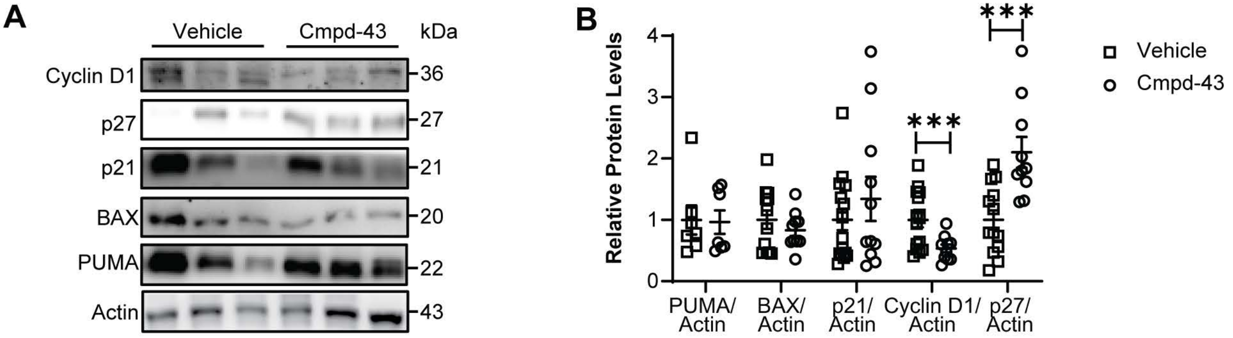

Supplementary Figure 6. Effect of pharmacological inhibition of PRL2 in inducible *Tp53*$^{-/-}$ model on P53 and FoxO targets. A) Representative western blot from inducible *Tp53*$^{-/-}$ and inducible *Tp53*$^{-/-}$ *Prl2*$^{-/-}$ derived thymic lymphomas to determine ERK activation. B) Quantification for (A), error bars represent the SEM, *Tp53*$^{-/-}$ n = 7-15, *Tp53*$^{-/-}$ *Prl2*$^{-/-}$n = 7-11. *** $p<0.001$
