# Supplementary material for: Inhibition of PRL2 Upregulates PTEN and Attenuates Tumor Growth in Tp53-deficient Sarcoma and Lymphoma Mouse Models
Source: Cancer Res Commun. 2024 Jan 2;4(1):5–17. doi: 10.1158/2767-9764.CRC-23-0308 (PMC10764713; doi:10.1158/2767-9764.CRC-23-0308)
Supplement: Figure S7 — Loss of PRL1 or PRL3 does not improve tumor-free survival of Tp53-null mice [file crc-23-0308-s07.pdf]

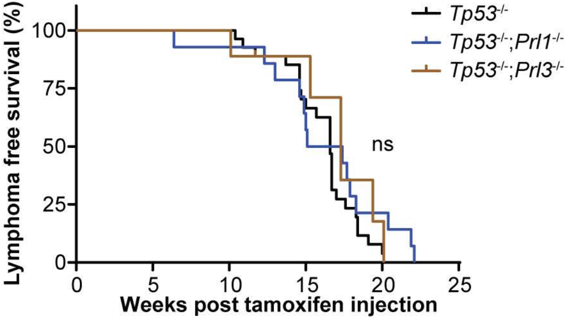

Supplementary Figure 7. Loss of PRL1 or PRL3 does not improve tumor-free survival of *Tp53-null* mice. Kaplan-Meier survival plot for thymic lymphoma free survival in tamoxifen inducible *Tp53*$^{-/-}$ (n = 27), *Tp53*$^{-/-}$; *Prl1*$^{-/-}$ (n = 14) and *Tp53*$^{-/-}$; *Prl3*$^{-/-}$ (n = 9) mice.
